# Effects and costs of a multi-component menstrual health intervention (MENISCUS) on mental health problems, educational performance, and menstrual health in Ugandan secondary schools: an open-label, school-based, cluster-randomised controlled trial

**DOI:** 10.1016/S2214-109X(25)00007-5

**Published:** 2025-04-24

**Authors:** Kate A Nelson, Stephen Lagony, Catherine Kansiime, Belen Torondel, Clare Tanton, Denis Ndekezi, Levicatus Mugenyi, Ratifah Batuusa, Christopher Baleke, Katherine A Thomas, Titus Ssesanga, Robert Bakanoma, Prossy Namirembe, Aggrey Tumuhimbise, Beatrice Nanyonga, Rodah Nambi, Edward Obicho, Denis Ssenyondwa, Daria Bucci, Sophie Belfield, Agnes Akech Ocen, Shamirah Nakalema, Connie Alezuyo, Fred Matovu, Stella Neema, Nambusi Kyegombe, Giulia Greco, John Jerrim, Chris Bonell, Janet A Seeley, Helen A Weiss

**Affiliations:** a MRC International Statistics and Epidemiology Group, London School of Hygiene & Tropical Medicine, London, UK; b Department of Disease Control, London School of Hygiene & Tropical Medicine, London, UK; c Department of Global Health and Development, London School of Hygiene & Tropical Medicine, London, UK; d Department of Public Health, Environments and Society, London School of Hygiene & Tropical Medicine, London, UK; e Medical Research Council/Uganda Virus Research Institute and London School of Hygiene & Tropical Medicine Uganda Research Unit, Entebbe, Uganda; f Africa Research Excellence Fund, MRC Unit The Gambia at The London School of Hygiene & Tropical Medicine, Fajara, The Gambia; g WoMena Uganda, Kampala, Uganda; h Ministry of Education and Sports, Kampala, Uganda; i Policy Analysis and Development Research Institute, School of Economics, Makerere University, Kampala, Uganda; j Department of Sociology and Anthropology, School of Social Sciences, Makerere University, Kampala, Uganda; k Institute of Education, University College London, London, UK

## Abstract

**Background:**

Menstrual health is a human rights issue, affecting many aspects of life including mental health, wellbeing, and education. We assessed the effectiveness and costs of a school-based, multi-component menstrual health intervention (MENISCUS) to improve mental health problems and educational performance among in-school adolescents.

**Methods:**

We conducted a parallel-arm, cluster-randomised trial in secondary schools in Wakiso and Kalungu districts in Uganda. Schools were eligible for inclusion if they had both male and female students; senior 1–4 classes; day or mixed day and boarding students; at least minimal water, sanitation, and hygiene (WASH) facilities; and enrolments of 50–125 female Senior 1 students in Wakiso district and 40–125 female Senior 1 students in Kalungu district. Schools were randomised (1:1) to the intervention or control condition, stratified by district and baseline mean school examination score. The intervention included creating action groups, strengthening teacher-delivered puberty education, distributing menstrual kits, supporting student-led drama skits, providing pain-management strategies, and improving school water and sanitation facilities. The control condition was provision of printed government menstrual health materials. Schools, participants, and implementors, including the study clinician who monitored adverse events, could not be masked to allocation status. Primary outcomes were mental health problems using the Strength and Difficulties Questionnaire (SDQ) Total Difficulties Score and independently assessed educational performance at individual level, assessed in all female participants at endline. We estimated cluster-intention-to-treat intervention effects using mixed-effects models accounting for school clustering and adjusted for randomisation strata and baseline school-level means of outcomes. The study was registered at the ISRCTN registry, ISRCTN45461276 and is completed.

**Findings:**

60 randomly selected schools (44 from Wakiso and 16 from Kalungu) were randomly assigned (30 per group) to the intervention or the control group, and none withdrew. Between March 21 and July 5, 2022, 3841 female students participated in baseline assessments (89·7% of those eligible) and between June 5 and Aug 22, 2023, 3356 participated in endline assessments (1666 in the control group and 1690 in the intervention group). Female participants had a median age of 16 years (IQR 15–16). At endline, there was no evidence of a difference in mental health problems (mean SDQ score, 10·8 in the intervention group *vs* 10·7 in the control group; adjusted mean difference [aMD] 0·05 [95% CI –0·40 to 0·50]) nor educational performance (mean z score, 0·20 in the intervention group *vs* 0·12 in the control group; aMD 0·05 [95% CI –0·10 to 0·19]), despite improvements to menstrual health. The annual implementation cost was US$85 per Senior 2 female student. One participant had a serious adverse event (severe anaemia secondary to excess vaginal bleeding), which was deemed to be possibly related to the intervention.

**Interpretation:**

Improving multiple dimensions of menstrual health in secondary schools in Uganda is important for health and human rights but is not sufficient to improve mental health or educational performance over 1 year.

**Funding:**

UK Foreign, Commonwealth and Development Office; Medical Research Council; Department of Health and Social Care; and Wellcome.

## Introduction

Menstrual health is defined as a state of physical, mental, and social wellbeing in relation to the menstrual cycle,^1^ and is a recognised human right.^2^ The definition reflects the multi-faceted nature of menstrual health, and the broad effects it can have on individuals’ health and wellbeing.^3^ Achieving menstrual health is essential to meet multiple Sustainable Development Goals and while there is growing commitment to improve menstrual health, there is little evidence to guide effective intervention.^4^

Qualitative studies have highlighted possible consequences of poor menstrual health on education, employment, physical and psychological health, and social participation.^3^ Shame, stigma, bullying, and distress during menstruation can contribute to reduced self-esteem, self-efficacy, and social participation, and to anxiety, depression, or conduct problems. Quantitative evidence for pathways linking menstrual health and mental health includes effects of menstrual-related hormonal fluctuations, dysmenorrhea, and access to menstrual management products on psychological distress, anxiety, and depressive disorders.^5^, ^6^ Multiple aspects of poor menstrual health can lead to school absence, including fear of blood leakage and unsupportive physical and social school environments.^3^ Interventions can improve menstrual-related knowledge and management, but few studies have quantified the effect of interventions on the sociocultural context of menstrual experience, and educational, health, and wellbeing outcomes.^7^, ^8^

**Research in context**

**Evidence before this study**

Menstrual health, defined as complete physical, mental, and social wellbeing in relation to the menstrual cycle, is recognised by WHO as a health and human rights issue. An integrated model of menstrual experience developed through a systematic review of qualitative studies illustrated pathways through which menstrual experience affects physical and psychological health, education, employment, and social participation. Two systematic reviews of the effectiveness of menstrual health interventions on health and social effects (2013) and education and psychosocial outcomes (2016) collectively identified five randomised controlled trials. None of these trials tested interventions addressing both the physical and psychosocial aspects of menstrual health, and the reviews concluded that there was insufficient evidence that menstrual health interventions improve school attendance or psychological wellbeing. The authors called for more rigorous evaluations of multi-component interventions, based on theories of change with improved measurement of core concepts. A review published in 2020 of intervention effects on menstrual-related school attendance and educational attainment again found limited and heterogenous evidence, and called for studies addressing pain management, the major reason for menstruation-related absenteeism. We searched PubMed, with no language restrictions, from Jan 1, 2015 (the end date of the 2016 systematic review) to April 6, 2024, for randomised controlled trials using the terms (“menstrua*” OR “menses” OR “menarche”) and (“school” OR “educ*” OR “student”). We identified two further school-randomised controlled trials. One found evidence that providing menstrual cups to secondary-school students in Kenya reduced incident herpes simplex virus type 2 infection but not HIV, school dropout rate, or pregnancy; the other found no effect of menstrual pad distributions or reproductive health education on school attendance in Kenyan primary schools, but positive effects on knowledge, attitudes, gender norms, and self-efficacy. Through colleagues, we identified three additional pre-prints of rigorous trials: a randomised controlled trial in Bangladesh which found a positive effect of a multi-component menstrual health programme on menstrual health, school attendance, and psychological wellbeing during menstruation (2021); a randomised controlled trial in The Gambia which found no evidence of an effect on school attendance or reproductive health outcomes (2023); and a randomised controlled trial in Uganda which found no evidence of an effect on school attendance following distribution of pads and education (2018).

**Added value of this study**

This randomised controlled trial adds to the limited evidence on the effectiveness of multi-component menstrual health interventions on educational and mental health outcomes and addresses priority research questions for improving menstrual health amidst growing global policy commitments. We found no evidence that the menstrual health intervention improved educational performance or reduced mental health problems among girls in secondary schools in Uganda but found evidence that the intervention improved multiple dimensions of menstrual health, including menstrual self-efficacy and use of effective pain management strategies. We found a beneficial intervention effect on attitudes towards menstruation among male and female participants. Qualitative findings suggested that the intervention was acceptable to, and valued by, participants. All resources used were costed to inform financial sustainability and cost-effectiveness analyses.

**Implications of all the available evidence**

Menstrual health is multidimensional, and multi-component interventions can lead to important improvements in the physical and social menstrual health environment in schools. Poor menstrual health is associated with poor mental health and school absenteeism, but menstrual health interventions might not be sufficient to lead to measurable improvements in these outcomes, amidst many other stronger influences.

In Uganda, only 35% of women aged 15–49 years report having an adequate physical and social environment for menstrual management.^9^ In 2016, our formative research among students aged 13–18 years in secondary schools in Wakiso District, Uganda, showed that poor menstrual health was associated with school absenteeism and poor wellbeing.^10^ We found a need for interventions to enable girls to better manage psychosocial and physical aspects of menstruation, and to include boys and teachers to improve the social environment. We then co-designed an intervention grounded in social cognitive theory (MENISCUS: Menstrual health Interventions, Schooling and Mental Health problems among Ugandan Students) with district and school-level stakeholders. We piloted the intervention in a pre-post study in two schools in Wakiso District, finding it feasible to deliver, acceptable, and valued by stakeholders. Following the intervention, there was improved school attendance and reduced mental health problems.^11^

To rigorously evaluate the effect of the intervention on education and health outcomes, we conducted the current cluster-randomised trial. Our hypothesis was that improved menstrual experiences and self-efficacy improves social participation, confidence, and school engagement and attendance during menstruation, leading to reduced internalising (eg, emotional symptoms and peer problems), and externalising (eg, conduct problems) mental health problems and improved educational performance. In this study, we report the effectiveness and costs of the MENISCUS menstrual health intervention on education and health outcomes in secondary schools in central Uganda.

## Methods

### Study design and participants

We conducted a parallel-arm, cluster-randomised controlled trial in 60 secondary schools in Wakiso and Kalungu districts, Uganda, from 2021 to 2024, with a process evaluation and economic and policy analyses.^12^ Schools were eligible if they had male and female students; senior 1–4 classes; day or mixed day and boarding students; at least minimal water, sanitation, and hygiene (WASH) facilities; and estimated enrolments of 50–125 female Senior 1 students in Wakiso District and 40–125 female Senior 1 students in Kalungu District. We excluded schools participating in existing menstrual health-related programmes and those exclusively for students with disabilities. We assessed schools for eligibility using the government's 2019 master list of education institutions. We checked the eligibility of all government schools and a random sample of private schools through phone calls and visits.^13^ We sought written, school-level consent from the headteacher or representative in a random sample of 60 schools confirmed eligible and willing to participate, stratified by government versus private schools and district.

We obtained enrolment lists from participating schools for the incoming Senior 2 class (academic year beginning January, 2022; mean age approximately 15·5 years). All female students enrolled in Senior 2 in a trial school and present during the survey period were eligible for the baseline survey and to receive the intervention. At endline, all female students present and enrolled in Senior 3, based on updated enrolment lists obtained as of March 31, 2023, were eligible for outcome assessments. We selected a simple random sample of 25 post-menarche female participants per school to self-complete a diary of their daily class attendance and menstrual cycle over approximately 3 months before the endline survey. If there were fewer than 25 post-menarche female participants, all were selected.

We selected a simple random sample of 15 male Senior 2 students per school to assess the intervention effect on knowledge and attitudes on menstrual health. Male participants enrolled in the same school at endline were eligible for outcome assessments. If there were fewer than 15 male students in a school, all were selected. Recruitment procedures occurred before random allocation.

Details of the informed consent procedures have been published previously.^12^ In brief, we sought electronic written informed assent from female students aged younger than 18 years with parental consent, and electronic written informed consent for those aged 18 years or older, immediately before the survey and after students watched an informational video. We sought separate parental consent and student assent to receive a menstrual cup, so that parents, guardians, and students could choose to participate in all trial activities except for receiving a cup. Consent and assent were sought before endline for female students newly enrolled in a trial school post baseline, using the same procedures in both groups.

Ethics approval for the trial was granted by the Uganda Virus Research Institute Research & Ethics Committee (reference GC/127/819), the Uganda National Council of Science and Technology (reference HS1525ES), and the London School of Hygiene & Tropical Medicine (reference 22952). Protocol amendments are detailed in appendix 1 (p 2). An independent Trial Steering Committee provided scientific guidance and monitored the progress of the trial. The Independent Data Monitoring and Ethics Committee (IDMEC) reviewed the trial recruitment and safety data and provided scientific guidance. The trial was prospectively registered at the ISRCTN registry, ISRCTN45461276, and is complete.

### Randomisation and masking

After the baseline survey, we randomised schools (1:1) to receive the MENISCUS intervention or optimised usual care. We stratified schools by district and baseline mean school examination score (above or below the median) and conducted covariate-constrained randomisation to minimise imbalance with respect to key factors: mean baseline examination score, past (2017–19) school examination scores, government or private school, mean baseline score for mental health problems and menstrual practice needs, and the number of female Senior 2 students enrolled in the trial (appendix 1 p 3). For each district, KAN and LM generated all possible random allocations, restricted to those meeting the specified stratification and balance criteria using the *cvcrand* R package, and then randomly selected 1000 of these allocations.

We conducted one randomisation ceremony per district, where school representatives pulled three numbered balls from an opaque bag (with replacement), forming a three-digit number corresponding to one of the 1000 allocations. A fourth ball was selected to decide which group would receive the intervention. Control group schools were offered the intervention after completion of endline assessments.

Schools, participants, implementors, and most study staff, including the study clinician who monitored adverse events, could not be masked to allocation status. The principal investigator (HAW), statistician (LM), and the Uganda National Examinations Board (UNEB) staff who independently administered the educational assessment were masked. To minimise assessment bias, surveys were self-completed by participants and co-ordinated by the research team, which was separate from the intervention implementor (NGO WoMena Uganda).

### Procedures

The MENISCUS theory of change and intervention was developed following formative and pilot studies (2015–18).^10^, ^11^, ^12^ The theory of change was grounded in social cognitive theory,^14^ with the intervention designed to positively reinforce observational learning and create a supportive environment for menstrual health, increasing participants’ self-efficacy to address their menstrual needs, and supporting behaviour change through improvements to the social and physical school environments.

The intervention consisted of: (1) training teachers to improve puberty education; (2) a student-led drama skit about menstrual health; (3) training selected student leaders, prefects, and teachers to deliver menstrual-health education sessions alongside the distribution of a menstrual kit containing reusable menstrual pads and an optional menstrual cup; (4) training on pain management, including provision of analgesics; and (5) improvements to school WASH facilities (described previously^12^). In each school, a Menstrual Health Action Group, consisting of teachers, students, or parents, was established to help coordinate and sustain the intervention. We used a train-the-trainer model, with the intervention implementor responsible for delivering central trainings to selected students and teachers who led school intervention activities. Details of the intervention delivery are given in appendix 1 (pp 14–16).

At baseline, we provided all 60 schools with optimised usual care: the research team distributed a copy of the government guidelines on menstrual hygiene management and sexuality education to the headteacher, and all male and female Senior 2 participants were given a copy of the government menstrual management reader.^15^, ^16^

We conducted the endline survey approximately 1 year after baseline survey and randomisation. All surveys were self-completed by participants on tablets at their school. The research team offered support if requested but otherwise did not view responses. Data were synced daily to a secure Open Data Kit Central server.

Female students who were newly enrolled in a trial school during the intervention year (identified through school enrolment lists as of March 31, 2023) completed a brief demographic survey at recruitment, before the endline survey. We distributed booklets to diary substudy participants approximately 12 weeks before the endline survey. We asked participants to complete them daily by shading boxes to answer six closed-ended questions on their school and class attendance and menstrual flow. Trial staff visited schools every 2–3 weeks to collect completed pages. Participants were permitted to retrospectively complete the diary within the current week. We assessed fidelity of implementation against prespecified indicators. Schools that met each indicator, based on a combination of observations, implementor logbooks, minutes, and school self-report, were considered to have implemented the intervention to a minimum intended level.

As part of the process evaluation, we conducted in-depth interviews with senior school staff members at baseline and endline (n=60), and with implementors (n=8) and school Menstrual Health Action Group members (n=30) during intervention delivery and at endline. We selected four case-study schools with varied baseline educational performance, menstrual cup consent, and school size. In each case-study school, we conducted three in-depth interviews with female students and teachers and three focus group discussions with female students, male students, and teachers, respectively, during intervention delivery and at endline. Participants were purposively sampled to ensure variation in demographic characteristics and degree of engagement with the intervention. We transcribed audio recordings for all the qualitative data verbatim, translated these into English (for those conducted in local dialect) and reviewed them for accuracy. We used thematic analysis to understand the potential mechanisms of impact and their interaction within context. A codebook was developed based on themes drawn from the topic guide and new themes drawn from detailed reading of the transcripts by social science team members. A team of four social scientists undertook the analysis. Coding was done independently with team members comparing their coding regularly to ensure consistency. After coding, the team organised the data by theme and thematic analytical memos were prepared.

We estimated all resources used for setting up and running each intervention component from a provider perspective, with a combination of top-down and micro-costing approaches. Financial and economic costs were identified and measured from project accounts and process evaluation data and valued using an adapted costing tool.^17^ Start-up costs were annuitised over their expected lifespans of 1·5 years and discounted at 7% and implementation costs discounted at 11% (the average interest rate during the start-up and implementation phases). Economic costs were used to reflect the value of non-financial costs (eg, donated menstrual cups). Research costs were excluded. Unit costs were calculated as the total annual incremental costs per student in Senior 2. Costs were incurred in Uganda shillings, adjusted to 2023 prices and converted to 2023 US$. Further details and sensitivity analyses are reported in appendix 1 (pp 19–21).

### Outcomes

The primary outcomes were educational performance and mental health symptoms among all female participants at endline. Educational performance was independently assessed by UNEB over 2 days during the baseline and endline periods, following standard national examination procedures. The baseline assessment covered the mathematics and biology secondary school syllabuses taught pre-intervention, and the endline assessment covered the mathematics, English, and biology syllabuses taught in Senior 2–3. The outcome was the mean z score for these subjects. Mental health problems were assessed using the Strengths and Difficulties Questionnaire (SDQ) Total Difficulties Score which is a dimensional measure of behavioural and emotional difficulties^18^ and has been widely used among adolescents in Africa.^19^ The possible range is 0–40, with higher scores indicating more problems. Secondary outcomes included dimensions of menstrual health and school attendance (table 1). Serious adverse events were defined as death, life-threatening event, in-patient hospitalisation or persistent or significant disability or incapacity, and were reported to the study clinician by trained designated teachers. Schools reported serious adverse events from the date of randomisation until Dec 31, 2023.

**Table 1:** Definitions of secondary outcomes

	**Definition**	**Analysis population**
Knowledge of puberty and menstruation	Number of factual items correct out of 9	All male and female participants (separately)
Attitudes and myths towards menstruation	Number of items with positive responses out of 3	All male and female participants (separately)
Adequate menstrual hygiene management	Use of only adequate menstrual materials that were appropriately cleaned or disposed of during last menstrual period*	Female participants who reported menstruating in the past 6 months
Menstrual experience	Menstrual Practice Needs Scale^20^ score (higher score indicates fewer unmet menstrual needs)†	Female participants who reported menstruating in the past 6 months
Effective pain management	Use of at least one predefined effective pain management method and no ineffective methods during last menstrual period	Female participants who reported menstruating in the past 6 months and reported pain during last menstrual period
Self-efficacy to address menstrual needs	Self-efficacy in Addressing Menstrual Needs Scale^21^ (higher scores indicate greater self-efficacy)	Female participants who reported menstruating in the past 6 months
Symptomatic urinary tract infection	One or more urogenital symptoms reported plus leucocyte esterase or nitrates with a urine Multistix 8 dipstick result (test done if more than symptom reported)	Female participants who reported menstruating in the past 6 months
School and class absence during menses	Odds of missing a full school day or lesson on period-days relative to non-period days, with period days defined as the days with flow plus the day before menstruation began	Diary substudy participants (female only)
School and class absence overall	Odds of missing a full school day or lesson on a school day	Diary substudy participants (female only)
Confidence in mathematics and science	Mean scores on the Trends in International Mathematics and Science Study, which measure confidence in mathematics and science scales	All female participants
Quality of life	Child Health Utility 9D Index (not included in current paper)	All female participants

* Defined as using a disposable pad or tampon that can be immediately disposed of; a reusable pad, cloth or towel, or homemade pad that is washed with water and soap and dried before use; or a menstrual cup that is boiled during or just before or after last menstrual period; and no inadequate materials reported.

† The Menstrual Practice Needs Scale score was calculated as the weighted average of: (1) the core items and school-specific items (given 75% weight) and (2) the relevant material-specific items (given 25% weight), since participants answer a different number of items depending on the materials reported.

### Statistical analysis

The sample size (60 schools) was estimated to provide 84% power to detect a target effect of a standardised mean difference (SMD) of 0·2 for continuous outcomes, assuming a harmonic mean of 60 female participants per school at endline, an intra-class correlation coefficient (ICC) of 0·05, and a two-sided significance level of 0·05. The effect size was based on the pilot study findings.^11^ Revised calculations were conducted in March, 2022, to reflect smaller school sizes after COVID-related school closures. With 60 schools, a harmonic mean of 40 female students per school at endline was estimated to provide 80% power to detect an SMD of 0·2.

The primary analysis was cluster-focused intention-to-treat, with schools analysed according to the group they were randomised to, using individual-level data from endline participants. Participants who dropped out were not followed and those who joined between baseline and endline were included. We adjusted analyses for randomisation strata and the baseline cluster-level mean of the outcome, where available, as fixed effects. All analyses accounted for clustering using a random effect for school.

For primary outcomes and continuous secondary outcomes, we estimated the intervention effect as the adjusted mean difference (aMD) and SMD at endline between groups using mixed-effects linear regression with 95% CIs. For count and binary outcomes, we estimated adjusted incidence rate ratios (aIRR) and adjusted odds ratios (aOR) using mixed-effects Poisson regression and mixed-effects logistic regression, respectively.

We estimated the aOR for school absence using mixed-effects logistic regression with random intercepts for school and student, using the diary data. We estimated intervention effects for absence on period days relative to non-period days as the interaction term between intervention group and period day. Period day was a binary variable defined apriori as a day of menses or the day before the first day of bleeding. We weighted this analysis by the inverse of the school-level sampling fraction, so results represented the female trial population.

We prespecified use of the Benjamini–Hochberg procedure to adjust the type 1 error for the two primary outcomes.^22^ For secondary outcomes, we made specific inferences for each individual null hypothesis and did not adjust the type 1 error.

We assessed effect-modification for primary outcomes by estimating p values for interaction terms by subgroup and trial group using the likelihood ratio test. Prespecified subgroup analyses were district, school ownership and predefined binary categories of school-level variables (baseline educational performance score, number of S2 female participants, and proportion of boarding students), and individual-level variables (age group, day or boarding status, socioeconomic status, and median baseline SDQ or UNEB score, respectively, for primary outcomes).

We also estimated the intervention effect within the closed cohort of female participants in the same school at baseline and endline, hypothesising that the intervention effects might be stronger than for the primary analysis population. As sensitivity analyses, we estimated intervention effects using cluster-level analyses and using independent estimating equations with robust standard errors to minimise bias in the presence of informative cluster size.

Statistical analyses were conducted using Stata 18.0, and costing analyses using Excel. The IDMEC approved the statistical analysis plan before the unmasking of trial data.

### Role of the funding source

The funder of the study had no role in study design, data collection, data analysis, data interpretation, or writing of the report.

## Results

Of 631 secondary schools listed, 504 did not meet the eligibility criteria (primarily due to size), 18 declined to participate, and one could not be contacted. We recruited 60 schools (44 from Wakiso and 16 from Kalungu), randomly selected from those confirmed eligible and willing to participate. Baseline participants were recruited from March 21 to July 5, 2022. Overall, 3841 (89·7%) of 4281 enrolled female Senior 2 students participated in the baseline survey (figure). Of these, 1699 (44·2%) participants or their parents gave consent and assent to receive a menstrual cup. 30 schools with 1921 students were randomly assigned to the control group and 30 schools with 1920 students were randomly assigned to the intervention group. All 60 schools participated in the endline survey (from June 5 to Aug 22, 2023). In total, 3356 female participants (1666 in the control group and 1690 in the intervention group), including 341 who had joined since baseline, contributed to the endline assessments (figure). The mean number of participants per school was 55·5 (SD 28·2, harmonic mean 40·5) in the control group and 56·3 (SD 30·4, harmonic mean 40·2) in the intervention group. Of the 3841 female baseline participants, 2991 (77·9%) were seen at endline in the same school (closed cohort). Of the 874 male baseline participants, 655 (74·9%) were seen at endline in the same school (appendix 1 p 8).

**Figure:**
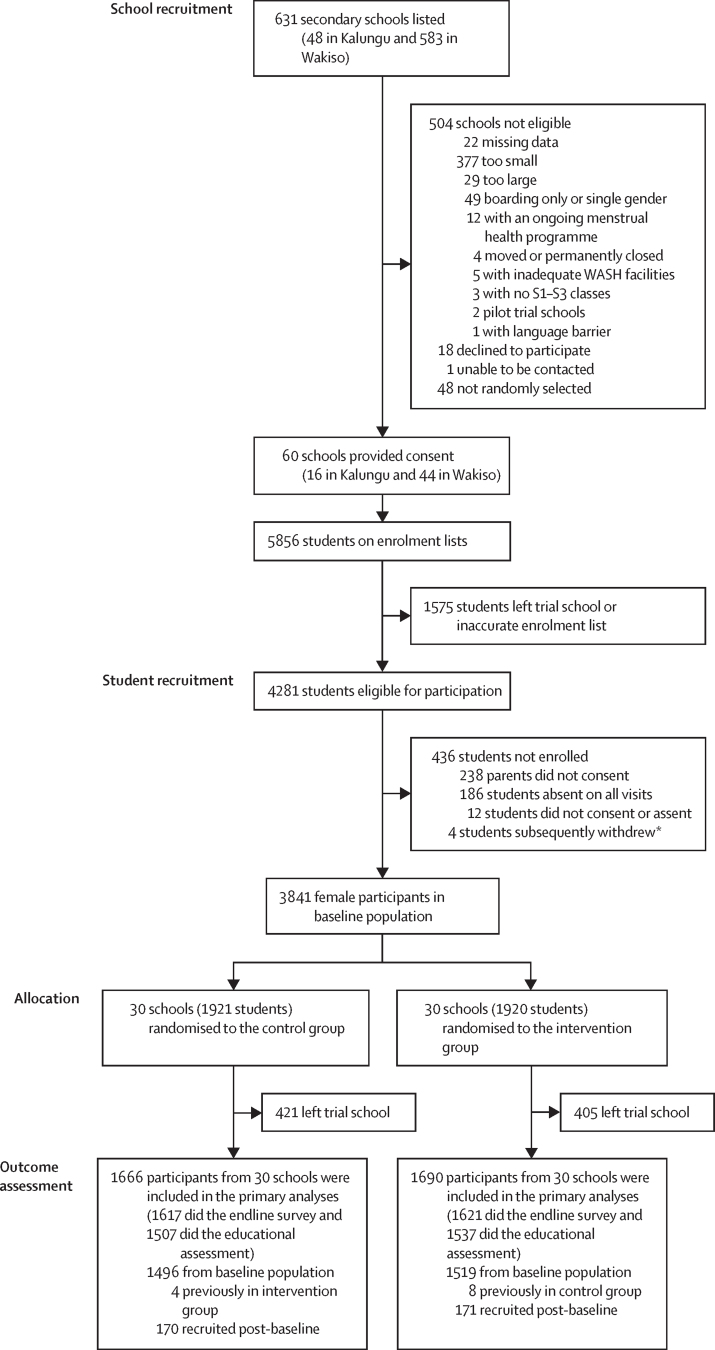
Trial profile At the allocation stage, the median school size of schools randomly assigned to the control group was 61·5 (IQR 43–76) which then decreased to 49·5 (34–70) at the outcome assessment stage. At the allocation stage, the median school size of schools assigned to the intervention group was 56 (IQR 43–74), which then decreased to 49·5 (34–74) at the outcome assessment stage. WASH=water, sanitation, and hygiene. *One from the control group and three from the intervention group.

Female participants had a median age of 16 years (IQR 15–16) at baseline. The mean baseline SDQ Total Difficulties Score was 12·2 (SD 5·6; Cronbach alpha 0·71). Baseline school and participant characteristics and baseline measures of the outcomes were balanced across groups (table 2; appendix 1 p 4).

**Table 2:** Baseline characteristics

		**Female participants**		**Male participants**	
		Control group (n=1921)	Intervention group (n=1920)	Control group (n=429)	Intervention group (n=445)
Number of participants		1921/3841 (50·0%)	1920/3841 (50·0%)	429/874 (49·1%)	445/874 (50·9%)
District					
	Kalungu	409 (21·3%)	450 (23·4%)	118 (27·5%)	119 (26·7%)
	Wakiso	1512 (78·7%)	1470 (76·6%)	311 (72·5%)	326 (73·3%)
Median age in years (IQR)		15 (15 to 16)	16 (15 to 16)	16 (15 to 17)	16 (15 to 17)
Age group, years					
	<15	190 (9·9%)	186 (9·7%)	28 (6·5%)	24 (5·4%)
	15	786 (40·9%)	757 (39·4%)	117 (27·3%)	103 (23·1%)
	16	702 (36·5%)	689 (35·9%)	137 (31·9%)	150 (33·7%)
	17	186 (9·7%)	222 (11·6%)	109 (25·4%)	118 (26·5%)
	≥18	57 (3·0%)	66 (3·4%)	38 (8·9%)	50 (11·2%)
Student type					
	Day	1058 (55·1%)	1066 (55·5%)	249 (58·0%)	254 (57·1%)
	Boarding	863 (44·9%)	854 (44·5%)	180 (42·0%)	191 (42·9%)
Religion					
	Catholic	593 (30·9%)	626 (32·6%)	154 (35·9%)	145 (32·6%)
	Protestant, Born Again, Seventh Day Adventist	719 (37·4%)	772 (40·2%)	151 (35·2%)	179 (40·2%)
	Muslim	597 (31·1%)	517 (26·9%)	122 (28·4%)	119 (26·7%)
	None or Other	12 (0·6%)	5 (0·3%)	2 (0·5%)	2 (0·4%)
Ethnicity					
	Muganda	1327 (69·1%)	1310 (68·2%)	310 (72·3%)	300 (67·4%)
	Non-Muganda	594 (30·9%)	610 (31·8%)	119 (27·7%)	145 (32·6%)
Primary caregiver*					
	Mother	1117 (58·1%)	1141 (59·4%)	211 (49·2%)	240 (53·9%)
	Father	472 (24·6%)	461 (24·0%)	168 (39·2%)	155 (34·8%)
	Self	5 (0·3%)	9 (0·5%)	3 (0·7%)	7 (1·6%)
	Other	327 (17·0%)	309 (16·1%)	47 (11·0%)	43 (9·7%)
Caregiver's education level					
	Primary or less	447 (23·3%)	457 (23·8%)	111 (25·9%)	110 (24·7%)
	Secondary or more	1156 (60·2%)	1121 (58·4%)	269 (62·7%)	277 (62·3%)
	Unknown	318 (16·6%)	342 (17·8%)	49 (11·4%)	58 (13·0%)
Household size, number of people					
	0–5	560 (29·2%)	619 (32·2%)	125 (29·1%)	143 (32·1%)
	6–7	630 (32·8%)	613 (31·9%)	138 (32·2%)	151 (33·9%)
	≥8	731 (38·1%)	688 (35·8%)	166 (38·7%)	151 (33·9%)
Meals eaten previous day					
	Three or more	599 (31·2%)	608 (31·7%)	151 (35·2%)	157 (35·3%)
	Two	955 (49·7%)	993 (51·7%)	226 (52·7%)	233 (52·4%)
	One or fewer	367 (19·1%)	319 (16·6%)	52 (12·1%)	55 (12·4%)
Socioeconomic position†					
	Lowest	642 (33·4%)	654 (34·1%)	144 (33·6%)	149 (33·5%)
	Medium	615 (32·0%)	659 (34·3%)	141 (32·9%)	149 (33·5%)
	Highest	664 (34·6%)	607 (31·6%)	144 (33·6%)	147 (33·0%)
Educational assessment z score‡		−0·25 (0·61)	−0·22 (0·62)	..	..
SDQ Total Difficulties Score		12·17 (5·60)	12·14 (5·61)	..	..

Data are mean (SD) or n (%). SDQ=Strengths and Difficulties Questionnaire.

* Participants were asked to select one primary caregiver; Other includes grandmother, grandfather, aunt, uncle, and other small categories.

† Socioeconomic position was derived using principal components analysis of participants' self-reported household assets and utilities.

‡ n=422 participants missing a baseline educational assessment score.

Of the 3356 female endline participants, 312 (9·3%) were missing UNEB assessment scores due to absence, 33 (1·0%) were missing a urinary tract infection (UTI) test result, and four (0·1%) were missing a Menstrual Practice Needs Scale score due to a survey error. Diary booklets were distributed to 1477 female participants from April 3, 2023. Of these, 1305 (88·4%) returned diaries, with a median of 69 (IQR 61–77) school day entries completed (appendix 1 p 9).

We found no evidence for an intervention effect on the mean educational performance score or the SDQ Total Difficulties Score. The mean z score for the educational assessment was 0·12 (SE 0·02) in the control group versus 0·20 (SE 0·02) in the intervention group, with no evidence for a difference (aMD 0·05 [95% CI –0·10 to 0·19]; SMD 0·06 [95% CI –0·12 to 0·24]; table 3). The mean SDQ Total Difficulties Score decreased from baseline to endline among participants in each group (12·2 to 10·7 in the control group and 12·1 to 10·8 in the intervention group), with no evidence of a difference between groups at endline (aMD 0·05 [95% CI –0·40 to 0·50]; SMD 0·01 [95% CI –0·07 to 0·09]). We found no intervention effect on the prespecified exploratory outcome of the SDQ internalising subscale (appendix 1 p 13).

**Table 3:** Intervention effects on primary and secondary outcomes

	**Control group**		**Intervention group**		**Effect estimates**		
	Number of participants	Mean (SE) or n (%)	Number of participants	Mean (SE) or n (%)	Adjusted effect estimate* (95% CI)	p value	SMD (95% CI)
**Primary outcomes**							
Educational assessment z score	1507	0·12 (0·02)	1537	0·20 (0·02)	aMD: 0·05 (−0·10 to 0·19)	0·56	0·06 (−0·12 to 0·24)
SDQ Total Difficulties Score	1617	10·73 (0·14)	1621	10·80 (0·14)	aMD: 0·05 (−0·40 to 0·50)	0·84	0·01 (−0·07 to 0·09)
**Female secondary outcomes**							
Knowledge score (out of 9)	1617	5·61 (0·03)	1621	6·15 (0·03)	aIRR: 1·10 (1·07 to 1·13)	0·0001	..
Attitudes score (out of 3)	1617	1·84 (0·02)	1621	2·20 (0·02)	aIRR: 1·20 (1·14 to 1·26)	<0·0001	..
Adequate menstrual hygiene management	1502	835 (55·6%)	1482	797 (53·8%)	aOR: 0·91 (0·76 to 1·08)	0·27	..
Menstrual Practice Needs Scale score	1503	2·28 (0·01)	1482	2·34 (0·01)	aMD: 0·09 (0·05 to 0·13)	0·0001	0·18 (0·09 to 0·27)
Self-Efficacy in Addressing Menstrual Needs Scale score	1506	64·08 (0·47)	1483	68·48 (0·48)	aMD: 4·95 (3·31 to 6·59)	<0·0001	0·27 (0·18 to 0·36)
Effective pain management	1268	845 (66·6%)	1219	919 (75·4%)	aOR: 1·50 (1·25 to 1·80)	<0·0001	..
Symptomatic urinary tract infection	1486	323 (21·7%)	1470	253 (17·2%)	aOR: 0·74 (0·54 to 1·00)	0·06	..
Confidence in mathematics	1617	1·60 (0·02)	1621	1·61 (0·02)	aMD: 0·01 (−0·05 to 0·07)	0·77	0·01 (−0·08 to 0·10)
Confidence in science	1617	1·99 (0·02)	1621	2·01 (0·01)	aMD: 0·02 (−0·03 to 0·08)	0·44	0·04 (−0·06 to 0·13)
**Male secondary outcomes**							
Knowledge score (out of 9)	314	5·44 (0·08)	341	5·73 (0·07)	aIRR: 1·04 (0·97 to 1·11)	0·27	..
Attitudes score (out of 3)	314	1·17 (0·06)	341	1·69 (0·06)	aIRR: 1·44 (1·26 to 1·64)	<0·0001	..
**Diary substudy outcomes**							
School absence†	36 035 days	10·5%‡	36 777 days	10·5%‡	aOR: 0·95 (0·73 to 1·24)	0·69	..
Days with class absence	36 035 days	15·3%‡	36 777 days	14·4%‡	aOR: 0·90 (0·71 to 1·16)	0·70	..
School absence during menstruation	5886 period days	13·5%‡	6246 period days	11·2%‡	aOR: 0·81 (0·62 to 1·05)	0·11	..
Days with class absence during menstruation	5886 period days	15·5%‡	6246 period days	14·4%‡	aOR: 0·97 (0·77 to 1·22)	0·77	..

aMD=adjusted mean difference. aOR=adjusted odds ratio. aIRR=adjusted incident rate ratio. SMD=standardised mean difference. SDQ=Strengths and Difficulties Questionnaire. Intracluster correlation coefficients: educational assessment=0·12; SDQ Total Difficulties=0·01.

* Adjusted for district, high or low school educational score, and the baseline cluster-level mean of the respective outcome measure where available (not included for symptomatic urinary tract infection and school or class absence outcomes; adequate menstrual hygiene management adjusted for use of only adequate material at baseline).

† Diary days from 651 participants in the intervention group and 652 participants in the control group.

‡ Diary substudy percentages are the proportion of days with the outcomes, weighted by the inverse school-level sampling fraction.

We found strong evidence of an intervention effect on most menstrual-related secondary outcomes, with small effect sizes (table 3). Compared with control group participants, those in the intervention group reported greater knowledge about puberty and menstruation, more positive attitudes towards menstruation, greater use of effective pain management, fewer unmet menstrual needs, and greater self-efficacy to manage menstruation. We found no evidence of an intervention effect on adequate menstrual hygiene management, defined as exclusive use of adequate materials that were disposed of or cleaned properly during their last menstrual period, and little evidence for an effect on the proportion with a symptomatic UTI. There was no evidence of a difference in female participants’ confidence in mathematics or science. Results were similar when restricted to the closed cohort (appendix 1 p 12). In the diary substudy, we found no evidence of an intervention effect on school or class attendance (on period days or overall; table 3).

Among male participants, we found evidence of a beneficial intervention effect on positive attitudes towards menstruation, and no evidence for an intervention effect on knowledge about puberty and menstruation (table 3).

All findings were robust to the alternative estimation methods (appendix 1 p 12). We found no evidence that the intervention effects differed by the prespecified subgroups (table 4).

**Table 4:** Effect-modification of intervention effect on primary outcomes

	**Educational performance**		**Mental health problems**	
	aMD (95% CI)	p_interaction_	aMD (95% CI)	p_interaction_
**District**				
Wakiso	0·15 (−0·15 to 0·46)	0·45	0·56 (−0·37 to 1·48)	0·22
Kalungu	0·01 (−0·17 to 0·19)	..	−0·11 (−0·61 to 0·40)	..
**Ownership**				
Private	−0·03 (−0·30 to 0·24)	0·47	0·19 (−0·56 to 0·95)	0·69
Government	0·09 (−0·10 to 0·28)	..	−0·004 (−0·56 to 0·55)	..
**School size**				
Below median	0·10 (−0·11 to 0·32)	0·53	0·33 (−0·41 to 1·06)	0·36
Above median	0·01 (−0·20 to 0·21)	..	−0·11 (−0·67 to 0·44)	..
**Proportion of participants boarding**				
<50%	0·08 (−0·12 to 0·27)	0·72	−0·15 (−0·75 to 0·45)	0·31
≥50%	0·02 (−0·20 to 0·24)	..	0·32 (−0·35 to 0·98)	..
**Age, years**				
<16	0·07 (−0·09 to 0·23)	0·45	−0·25 (−0·86 to 0·37)	0·18
≥16	0·03 (−0·13 to 0·19)	..	0·28 (−0·30 to 0·86)	..
**Day or boarding student**				
Boarding	0·05 (−0·11 to 0·22)	0·79	−0·29 (−0·91 to 0·33)	0·33
Day	0·04 (−0·13 to 0·20)	..	0·13 (−0·54 to 0·79)	..
**Socioeconomic position**				
Below median	0·05 (−0·11 to 0·22)	0·81	−0·09 (−0·71 to 0·52)	0·51
Above median	0·04 (−0·13 to 0·21)	..	0·17 (−0·41 to 0·74)	..
**Baseline educational assessment score**				
Below median	0·05 (−0·13 to 0·22)	0·94	..	..
Above median	0·05 (−0·12 to 0·22)	..	..	..
**Baseline SDQ Total Difficulties Score**				
Below median	..	..	−0·08 (−0·71 to 0·56)	0·63
Above median	..	..	0·10 (−0·45 to 0·64)	..

aMD=adjusted mean difference. SDQ=Strengths and Difficulties Questionnaire. Subgroups for school size, age, socioeconomic position, educational assessment score, and SDQ determined by the median value.

The intervention implementor delivered all planned district-level training sessions within 3·5 months of randomisation, attended by 29 of 30 intervention schools. Menstrual health kits were distributed in all schools by 6 months after randomisation. The final implementor-led intervention activity (training of drama skit facilitators) was completed by 9 months after randomisation. Fidelity of school-led activities varied across components (appendix 1 pp 17–18). Overall, 20 of 28 schools with complete process data implemented all intervention components to the minimum intended level (excluding the availability of analgesics, which we were unable to measure). To our knowledge, 12 of the 30 control schools reported some menstrual health-related activities such as pad distributions during trial follow-up.

Qualitative findings indicated that the intervention was widely accepted among school communities (students, teachers and parents) and positively affected menstrual experiences at schools. Female trial participants reported greater confidence in managing menstruation due to improvements to the social and physical school environments, such as the WASH facilities and access to menstrual products. Menstrual Health Action Groups were reported to successfully engage the school community in activities (especially WASH and puberty education) and were perceived to be most successful when they had active student involvement. Staff turnover and motivation were challenging in some schools, affecting implementation. Provision of free reusable menstrual products alleviated participants’ and parents’ stress regarding acquiring disposable pads. Conversely, some participants reported not using the reusable products at school due to having to wash and dry them, with fears about washing menstrual blood and embarrassment of carrying a used pad. Education sessions, drama skits, and distribution of analgesics were perceived to have normalised menstruation, provided information about pain and menstrual management, and addressed misconceptions about painkillers. Key challenges were maintenance of WASH facilities and access to painkillers. The central involvement of male students was seen as key to intervention success by improving interactions among male and female students, leading to more support from male students and reduced stigma**.** Additional qualitative findings will be reported separately.

The incremental cost of setting up the intervention was US$40 990. The annual implementation cost was $181 503, equivalent to a unit cost of $6050 per school, $44 per Senior 2 student (male and female), or $85 per Senior 2 female student. The largest cost drivers in the implementation were supplies (33%) and salaries (29%; appendix 1 p 20).

Three participants in the intervention group and two participants in the control group had serious adverse events. One of the serious adverse events (severe anaemia secondary to excess vaginal bleeding, treated successfully) was possibly related to the intervention (appendix 1 p 11).

## Discussion

To our knowledge, this is one of the first randomised controlled trials to evaluate the effect of a multi-component menstrual health intervention on educational performance and mental health problems. We found evidence of effects on menstrual health outcomes including pain management, menstrual self-efficacy, and attitudes, but these were insufficient to impact the primary educational and mental health outcomes over 1 year.

Few evaluations of menstrual health interventions have included mental health outcomes.^8^ The lack of effect of our intervention on mental health problems is consistent with results from a school-based randomised controlled trial in Bangladesh, which found no intervention effect on psychological wellbeing measured by the Mental Health Index,^23^ and results from a quasi-randomised trial in Uganda which found no effect on psychosocial outcomes including the SDQ.^24^

The lack of intervention effect on mental health might be due to multiple factors. Although we found strong evidence of an intervention effect on almost all dimensions of menstrual health measured, these effects were modest and might have been insufficient to lead to a measurable effect on mental health problems. A longer duration between the intervention implementation and endline survey, to allow for participants to have repeated positive menstrual experiences, might also be needed to affect mental health. Given biopsychosocial links between menstrual health and mental health,^5^ the lack of effect probably also reflects the multiple causes of mental health problems among adolescents. A systematic review of brief, school-based counselling interventions which directly targeted mental health found heterogeneous but small positive effects on mental health or wellbeing, underscoring the challenge of improving mental health through school-based programmes.^25^ Finally, the improvement in mental health problems over time in both groups in our trial indicates a possible beneficial effect of participating in the research activities.

This is the first trial of a menstrual health intervention to include an educational performance outcome, in addition to absenteeism. We hypothesised that education performance would be improved by addressing menstrual factors associated with school absence and reduced engagement (pain, lack of menstrual products, poor WASH facilities, and stigma or behavioural restrictions).^3^, ^12^ The lack of evidence of an intervention effect on either absenteeism or performance adds to the limited body of evidence. A systematic review found moderate but non-significant effects on school attendance associated with menstrual product distribution interventions, and low levels of menstrual-related absenteeism overall,^8^ and a randomised controlled trial in Kenyan schools found no effect of providing menstrual cups and menstrual education on school absenteeism or dropout rate.^26^ The lack of association might be partly due to the small number of school-days during menstruation (0–5 per month) and challenges measuring school attendance.^27^

The quality of school-led implementation was encouraging amidst COVID-related challenges and staff turnover. The lack of intervention effect is unlikely to be due to poor implementation given the adequate fidelity observed. Qualitative data showed the intervention to be highly valued by school staff and students. The reported menstrual health-related activities in some control schools might have attenuated the observed intervention effects on secondary menstrual health outcomes and demonstrates the added value of multi-component interventions that go beyond product provision.

A strength of our study is the alignment of the intervention and the theory of change with both the definition of menstrual health^1^ and the integrated model of menstrual experience.^3^ Our intervention was innovative in its focus on improving menstrual self-efficacy and the social environment, including attitudes among boys, and had an effect on almost all dimensions of menstrual health in the model.^3^ The lack of effect on the proportion of participants reporting using adequate menstrual materials that were disposed of or cleaned properly is consistent with our qualitative findings that reusable pads were less convenient to use at school than disposable pads. These findings highlight the importance of participant-centred interventions that improve perceived menstrual needs, beyond promoting objectively defined measures of good menstrual management.

Additional strengths included a representative and heterogeneous sample of large secondary schools in two Ugandan districts, supporting generalisability to this population. The acceptability of the intervention was also reflected in the minimal response bias with a high proportion of consent and assent at the school, parent, and student levels. We minimised assessment bias with independent assessment of educational performance, self-completed surveys, and collection of baseline data before randomisation. We minimised measurement bias using validated up-to-date tools for menstrual-related outcomes when possible. Measuring school attendance is challenging and we used recommended data collection methods^27^ validated in this setting against observational spot-checks for attendance.^11^

A limitation was the timing of the intervention roll-out and endline assessment. The intervention took longer than anticipated to be fully delivered, with delays largely due to over-burdened schools following lengthy COVID-19-related school closures. These closures also meant that the school-led intervention implementation was split over two academic years, leading to interruptions during examination periods and holidays. Previous studies have shown that menstruators often take several months to become comfortable using the menstrual cup, so we may not have captured the full potential benefit.^28^ The dynamic school environment, with students and staff leaving and joining throughout the academic year, meant that not all students received the full possible exposure to the intervention. Our trial design allowed us to capture the effects of an intervention when delivered in this real-world setting, but this turnover has implications for longer-term sustainability of train-the-trainer intervention models which we will explore further in the process evaluation.

We used the SDQ to assess mental health problems due to its widespread use including among adolescents in sub-Saharan Africa. Although it can be used to assess risk of emotional and conduct disorders, it might not directly capture some aspects of mental health problems relevant to menstrual health, such as depression and anxiety.^19^ However, there are few alternative validated tools to assess mental health among adolescents at the population-level in sub-Saharan Africa.^29^ It is possible that more targeted interventions are needed to address menstrual cycle disorders and severe dysmenorrhea, which might have stronger effects on mental health.

The cost of the MENISCUS intervention per Senior 2 female student (US$85) exceeded that of a comparable trial in Kenya, in which the estimated annual cost of providing menstrual kits (cup or disposable pads and soap) and puberty education training was US$34 per direct recipient.^30^ The higher cost of the MENISCUS intervention is likely to be attributable to the inclusion of additional components. Moreover, some intervention components were designed to benefit the entire school community, which would substantially reduce the cost per beneficiary if considered.

In a context of growing advocacy, policy, and public interest around menstrual health, it is crucial to build the evidence base for what works to address substantial unmet menstrual health needs globally. We provide novel evidence for the effectiveness of a multi-component menstrual health intervention. Although the intervention achieved modest improvements in multiple dimensions of menstrual health, these were not sufficient to impact mental health or educational performance as widely hypothesised. Further research is needed to strengthen interventions to improve adolescents’ menstrual health as a human rights issue, and to directly address their mental health and educational needs.

### Contributors

### Equitable partnership declaration

### Data sharing

The de-identified individual participant data that underlie the results reported in this Article are available indefinitely on request from the London School of Hygiene & Tropical Medicine Data Compass at https://doi.org/10.17037/DATA.00003822, along with the codebook, informed consent documents, and qualitative interview guides. Participants gave informed consent for their data to be published after de-identification. The statistical analysis plan is publicly available on the trial registration page: https://www.isrctn.com/ISRCTN45461276.

## Declaration of interests

We declare no competing interests.

## Supplementary Materials

Supplementary appendix 1

Equitable Partnership Declaration
