# Supplementary material for: Effects and costs of a multi-component menstrual health intervention (MENISCUS) on mental health problems, educational performance, and menstrual health in Ugandan secondary schools: an open-label, school-based, cluster-randomised controlled trial
Source: Lancet Glob Health. 2025 Apr 24;13(5):e888–99. doi: 10.1016/S2214-109X(25)00007-5 (PMC12041187; doi:10.1016/S2214-109X(25)00007-5)
Supplement: Supplementary appendix 1 [file mmc1.pdf]

THE LANCET
Global Health

Supplementary appendix 1

This appendix formed part of the original submission and has been peer reviewed. We post it as supplied by the authors.

Supplement to: Nelson KA, Lagony S, Kansiime C, et al. Effects and costs of a multi-component menstrual health intervention (MENISCUS) on mental health problems, educational performance, and menstrual health in Ugandan secondary schools: an open-label, school-based, cluster-randomised controlled trial. *Lancet Glob Health* 2025; **13:** e888–99.

Supplementary material

Table of Contents

1. Details on methods 2
- 1.1. Changes to protocol and secondary outcome measures 2
- 1.2. Details on modifications due to extenuating circumstances (CONSERVE guidelines) 2
- 1.3. Restricted randomisation criteria used 3
- 1.4. Benjamini Hochberg procedure 3

2. Additional trial description and profiles 4
- 2.1. Table: Baseline school characteristics by arm 4
- 2.2. Table: Baseline measures of secondary outcomes by arm 4
- 2.3. Table: Characteristics of female endline population by arm 5
- 2.4. Table: Characteristics of baseline female population by endline enrollment status and arm 6
- 2.5. Table: Characteristics of baseline male population by endline enrollment status 7
- 2.6. Figure: Male participant profile 8
- 2.7. Figure: Diary sub-study participant profile 9
- 2.8. Figure: Distribution of SAMNS and MPNS scores by arm 10

3. Safety reporting 11
- 3.1. Table: Serious AEs and non-serious AEs 11

4. Additional analyses (pre-specified) 12
- 4.1. Table: Intervention effects among female participants in the closed cohort 12
- 4.2. Table: Sensitivity analyses 12
- 4.3. Table: Intervention effect on the SDQ internalising sub-scale 13

5. Intra-cluster correlation estimates 13
- 5.1. Table: Intra-cluster correlation by arm and overall for continuous and binary outcomes 13

6. Implementation fidelity 14
- 6.1. Table: MENISCUS intervention reported according to the TIDieR Framework 14
- 6.2. Table: Fidelity indicators and definitions 17
- 6.3. Table: Fidelity of intervention implementation by district 18

7. Costing methods, detailed results, and sensitivity analysis 19
- 7.1. Methods 19
- 7.2. Table: Activities, resource use and costing sources for each cost component of costing analysis 20
- 7.3. Table: Cost of setting up and running the MENISCUS intervention 20
- 7.4. Table: Implementation cost per intervention component (USD) 21
- 7.5. Table: Total cost per beneficiary 21
- 7.6. Sensitivity analysis 21
- 7.7. Cost comparison 21

1. Details on methods

1.1. Changes to protocol and secondary outcome measures

A detailed description of all protocol amendments is included on pages 2-3 of the trial protocol version 6.0. The secondary outcome on prevalence of bacterial vaginosis (BV), vaginal yeast and urinary tract infections (UTIs) among symptomatic girls was revised to the prevalence of UTIs only among symptomatic girls. This change was approved by the TSC and IDMEC to remove the time burden on schools, participants, and data collection teams. Additional changes to the secondary outcome measures, made prior to unblinding and reflected in the Statistical Analysis Plan, are listed below.

Table 1.1 Summary of changes made to secondary outcome measures and rationale:

Outcome (protocol v4.0)	Measure (protocol v4.0)	Revised measure (protocol v7.0)	Rationale
1. Knowledge of puberty and menstruation; attitudes towards menstruation	Proportion answering all knowledge questions correctly.	Number of knowledge items answered correctly (out of 9)	To make full use of the count data and avoid a cut-off
	Proportion answering all questions on myths correctly	*Removed and combined with below*	Not measured as separate construct
	Proportion with "good responses" on attitudes	Number of myths and attitude items with positive responses (out of 3)	To make full use of the count data and avoid a cut-off
2. Menstrual practices at last menstrual period (LMP)	Proportion using manufactured methods only at LMP	*Removed*	Use of adequate materials incorporated into below measure
	Proportion correctly washing and drying re-usable pads and/or menstrual cups at LMP	*Removed*	Denominator likely to be influenced by the intervention
	Proportion with 'adequate MHM at school' at LMP (Self-reported use of clean materials to absorb/collect blood, changed privately, safely, hygienically, and as often as needed)	Proportion using only adequate menstrual materials that are appropriately cleaned or disposed of at their last menstrual period	Restricted to use and disposal of clean materials to limit overlap with MPNS outcome
3. Knowledge and practices of pain management during LMP	Proportion knowing 4 or more effective pain management methods (Use of painkiller, drinking water, using water bottle, exercise, relaxing, foods with lots of water)	*Removed*	Reduced number of measures to focus on practice of pain management which implies knowledge
	Proportion who used an effective pain management method	Proportion who used an effective pain management method (at least 1 effective method and no ineffective methods)	Clarified that no ineffective methods could be reported
5. Quality of life and happiness	Mean CHU9D score	CHU9D score calculated as a weighted sum using adolescent utility tariffs	Clarified that score would be calculated using validated weights in our population
	Self-reported measure of happiness	*Removed*	Reduced number of measures; moved to exploratory outcome

1.2. Details on modifications due to extenuating circumstances (CONSERVE guidelines)

Extenuating circumstances:

The trial commenced during the COVID-19 pandemic, with national school closures from March 2020 to January 2022. School recruitment and parental consent began in 2021 and extended into 2022. Student assent and the baseline survey began in March 2022, after schools reopened. Fieldwork was not interrupted by the COVID-19 closures.

During intervention delivery in November 2022, the Ugandan Ministry of Education and Sports ended Term 3 approximately two weeks early due to an Ebola outbreak.

Important modifications:

These extenuating circumstances had effects on the trial environment and conduct as described below. We implemented the following mitigating strategies:

- Uncertain class sizes and reduced school enrolment when schools reopened: The sample size was increased from 48 to 60 schools. The eligibility criterium around school size was based on data as of January 2020 and actual school sizes varied. (Amended protocol v1.3, May 2021)
- Restrictions on parental information and consent meetings at schools, and difficulties reaching parents of students: Parents who were unable to attend information and consent meetings were contacted by phone (through the school Head Teacher) and verbal informed consent was sought. (Amended protocol v2.0, August 2021)
- Delays to student recruitment and the baseline survey: Parental consent and student recruitment, originally planned for Term 3 of Senior 1, was delayed. This meant that the 1-year intervention period spanned two academic years (AY) and included the holiday between AY 2022 (when participants were in Senior 2) and AY 2023 (when participants were in Senior 3).
- Reduced curriculum covered for the baseline UNEB assessment: UNEB staff contacted head teachers of trial schools in August 2021 to confirm the curriculum topics which had been covered prior to the lockdown in Senior 1. The baseline assessment covered a smaller amount of material than initially planned. Impact on the trial was minimal as the assessment was used for baseline adjustment not outcome measurement.
- Students older in age for their class year: Students were one year older, on average, for Senior 2. This did not directly affect the intervention content, which was originally designed to be delivered to post-menarche students.
- Increased burden on school staff: It is possible schools' engagement with the intervention activities was reduced from what it would have been prior to COVID-19 closures. However, the intervention was still implemented with adequate fidelity by most schools.

Responsible parties

All modifications to the trial design and conduct were discussed among the trial team and reviewed and approved by the TSC and IDMEC. Where applicable, formal amendments to the trial protocol were approved by the Ugandan and LSHTM ethics committees.

1.3. Restricted randomisation criteria used

In addition to stratification by high/low UNEB baseline assessment score and district, the following restriction criteria were used to minimise imbalance in key baseline characteristics within each district:

- Relative mean difference ≤25%: UNEB maths assessment score; UNEB biology assessment score; SDQ Total Difficulties score; MPNS score; proportion of female participants who are boarding students; past (2017-2019) exam performance in English, Math, Biology; proportion of participants with consent and assent to receive a menstrual cup
- Relative mean difference ≤20%: Number of S2 female participants
- Absolute difference ≤2 schools: Government ownership

1.4. Benjamini Hochberg procedure

We pre-specified use of the Benjamini-Hochberg (BH) procedure to adjust the Type 1 error given the two primary outcomes.[1] The procedure states that for the two primary outcomes considering a false discovery rate (FDR) of 0.05: 1) if the larger p-value ≤0.05, then there is evidence for both end points; 2) if the larger p-value >0.05, then the smaller p-value should be ≤0.025 to show evidence for that endpoint, and 3) if the larger p-value >0.05 and the smaller p-value>0.025, then there is no evidence for both endpoints.

2. Additional trial description and profiles

2.1. Table: Baseline school characteristics by arm

School characteristics	Control arm	Intervention arm
Number of schools	30 (50·0%)	30 (50·0%)
Mean female participants per school	64·0 (31·2)	64·0 (33·5)
District		
Kalungu	8 (26·7%)	8 (26·7%)
Wakiso	22 (73·3%)	22 (73·3%)
Ownership		
Government	9 (30·0%)	10 (33·3%)
Private	21 (70·0%)	20 (66·7%)
Mean % of female participants who are boarding students	43·8 (27·5)	43·8 (26·6)
Mean % of female participants with consent and assent to receive a menstrual cup	48·0 (26·1)	47·2 (22·6)
At least one toilet cubicle which is single-sex for girls, private, and functional with a waste bin	6 (20·0%)	8 (26·7%)

Data are n (%), mean (SD), or n/N (%)

2.2. Table: Baseline measures of secondary outcomes by arm

	Control arm		Intervention arm	
**Female secondary outcome measures**	N	Mean (SD) / n (%)	N	Mean (SD) / n (%)
Knowledge score (out of 9)	1921	5·06 (1·49)	1920	5·03 (1·50)
Attitudes score (out of 3)	1921	1·48 (0·94)	1920	1·48 (0·94)
Only adequate menstrual materials used at LMP*	1638	1509 (92·1%)	1643	1500 (91·3%)
MPNS score	1624	2·12 (0·52)	1628	2·10 (0·52)
SAMNS score	1638	61·13 (18·63)	1643	60·48 (19·37)
Pain during LMP	1638	1222 (74·6%)	1643	1204 (73·3%)
Effective pain management	1222	751 (61·5%)	1204	766 (63·6%)
Confidence in maths scale score	1921	1·68 (0·67)	1920	1·69 (0·67)
Confidence in science scale score	1921	2·07 (0·60)	1920	2·05 (0·61)
**Male secondary outcome measures**				
Knowledge score (out of 9)	429	4·75 (1·46)	445	4·93 (1·50)
Attitudes score (out of 3)	429	0·87 (0·89)	445	0·92 (0·86)

LMP = last menstrual period; MPNS = Menstrual Practice Needs Scale, SAMNS = Self Efficacy in Addressing Menstrual Needs Scale
*Does not include washing and drying of materials which is part of the outcome definition
** Denominator for effective pain management outcome

2.3. Table: Characteristics of female endline population by arm

Characteristics	Arm allocation	
	Control arm	Intervention arm
Number of participants	1,666 (49·6%)	1,690 (50·4%)
District		
Kalungu	342 (20·5%)	395 (23·4%)
Wakiso	1,324 (79·5%)	1,295 (76·6%)
Age in years	16·5 (0·9)	16·6 (1·0)
Age group		
<15 years	6 (0·4%)	12 (0·7%)
15 years	153 (9·5%)	130 (8·0%)
16 years	702 (43·4%)	710 (43·8%)
17 years	594 (36·7%)	551 (34·0%)
≥18 years	162 (10·0%)	218 (13·4%)
Student type		
Day	821 (54·4%)	846 (55·0%)
Boarding	688 (45·6%)	692 (45·0%)
Religion		
Catholic	534 (32·1%)	544 (32·2%)
Protestant/Born Again/SDA	607 (36·4%)	693 (41·0%)
Muslim	518 (31·1%)	449 (26·6%)
None/Other	7 (0·4%)	4 (0·2%)
Ethnicity		
Muganda	1,156 (69·4%)	1,148 (67·9%)
Non Muganda	510 (30·6%)	542 (32·1%)
Primary caregiver		
Mother	979 (58·8%)	985 (58·3%)
Father	412 (24·7%)	436 (25·8%)
Self	4 (0·2%)	6 (0·4%)
Other	271 (16·3%)	263 (15·6%)
Household size		
0-5 people	482 (28·9%)	534 (31·6%)
6-7 people	550 (33·0%)	557 (33·0%)
≥8 people	634 (38·1%)	599 (35·4%)
Meals eaten previous day		
Three or more	475 (31·5%)	513 (33·4%)
Two	748 (49·6%)	779 (50·7%)
One or fewer	286 (19·0%)	246 (16·0%)
Relative socioeconomic position		
Lowest	482 (33·0%)	497 (33·8%)
Medium	464 (31·8%)	512 (34·8%)
Highest	515 (35·2%)	462 (31·4%)

2.4. Table: Characteristics of baseline female population by endline enrollment status and arm

	Control arm			Intervention arm		
	Endline status			Endline status		
	Seen at endline	Not seen at endline	p-value	Seen at endline	Not seen at endline	p-value
N	1,500 (78.1%)	421 (21.9%)		1,515 (78.9%)	405 (21.1%)	
District			0.90			0·51
Kalungu	313 (20.9%)	96 (22.8%)		352 (23.2%)	98 (24.2%)	
Wakiso	1,187 (79.1%)	325 (77.2%)		1,163 (76.8%)	307 (75.8%)	
Age in years	15.52 (0.91)	15.64 (0.98)		15.57 (0.98)	15.72 (0.97)	
Age group			0.10			0·03
<15 years	148 (9.9%)	42 (10.0%)		156 (10.3%)	30 (7.4%)	
15 years	640 (42.7%)	146 (34.7%)		615 (40.6%)	142 (35.1%)	
16 years	536 (35.7%)	166 (39.4%)		524 (34.6%)	165 (40.7%)	
17 years	135 (9.0%)	51 (12.1%)		170 (11.2%)	52 (12.8%)	
≥18 years	41 (2.7%)	16 (3.8%)		50 (3.3%)	16 (4.0%)	
Student type			0.40			0·14
Day	818 (54.5%)	240 (57.0%)		828 (54.7%)	238 (58.8%)	
Boarding	682 (45.5%)	181 (43.0%)		687 (45.3%)	167 (41.2%)	
Religion			0.02			0·09
Catholic	483 (32.2%)	110 (26.1%)		498 (32.9%)	128 (31.6%)	
Protestant/Born Again/SDA	546 (36.4%)	173 (41.1%)		618 (40.8%)	154 (38.0%)	
Muslim	464 (30.9%)	133 (31.6%)		397 (26.2%)	120 (29.6%)	
None/Other	7 (0.5%)	5 (1.2%)		2 (0.1%)	3 (0.7%)	
Ethnicity			0.20			0·84
Muganda	1,043 (69.5%)	284 (67.5%)		1,033 (68.2%)	277 (68.4%)	
Non Muganda	457 (30.5%)	137 (32.5%)		482 (31.8%)	128 (31.6%)	
Primary caregiver			0.65			0·05
Mother	879 (58.6%)	238 (56.5%)		889 (58.7%)	252 (62.2%)	
Father	368 (24.5%)	104 (24.7%)		384 (25.3%)	77 (19.0%)	
Self	4 (0.3%)	1 (0.2%)		6 (0.4%)	3 (0.7%)	
Other	249 (16.6%)	78 (18.5%)		236 (15.6%)	73 (18.0%)	
Household size			0.69			0·42
0-5 people	432 (28.8%)	128 (30.4%)		481 (31.7%)	138 (34.1%)	
6-7 people	495 (33.0%)	135 (32.1%)		495 (32.7%)	118 (29.1%)	
≥8 people	573 (38.2%)	158 (37.5%)		539 (35.6%)	149 (36.8%)	
Meals eaten previous day			0.97			0·02
Three or more	472 (31.5%)	127 (30.2%)		503 (33.2%)	105 (25.9%)	
Two	744 (49.6%)	211 (50.1%)		766 (50.6%)	227 (56.0%)	
One or fewer	284 (18.9%)	83 (19.7%)		246 (16.2%)	73 (18.0%)	
Relative socioeconomic position			0.33			0·25
Lowest	482 (32.1%)	160 (38.0%)		500 (33.0%)	154 (38.0%)	
Medium	483 (32.2%)	132 (31.4%)		528 (34.9%)	131 (32.3%)	
Highest	535 (35.7%)	129 (30.6%)		487 (32.1%)	120 (29.6%)	
Baseline MPNS score	2.12 (0.52)	2.03 (0.54)	0.006	2.09 (0.52)	2.04 (0.54)	0·12
Baseline SAMNS score	60.99 (18.72)	58.92 (19.35)	0.10	60.67 (19.30)	58.19 (20.05)	0·03
Baseline educational assessment z-score	0.01 (0.83)	-0.12 (0.78)	0.01	0.05 (0.84)	-0.12 (0.80)	0·03
Baseline SDQ Total Difficulties score	11.85 (5.51)	13.32 (5.77)	<0.001	12.02 (5.64)	12.59 (5.49)	0·05

Data are mean (SD) or n (%); SDQ = Strengths and Difficulties Questionnaire

2.5. Table: Characteristics of baseline male population by endline enrollment status

	Endline status		
	**Seen at endline**	**Not seen at endline**	**p-value for difference**
**N**	**658 (75·3%)**	**216 (24·7%)**	
District			
Kalungu	181 (27·5%)	56 (25·9%)	0·68
Wakiso	477 (72·5%)	160 (74·1%)	
Age in years	16·06 (1·14)	16·25 (1·12)	
Age group			
<15 years	43 (6·5%)	9 (4·2%)	0·18
15 years	175 (26·6%)	45 (20·8%)	
16 years	213 (32·4%)	74 (34·3%)	
17 years	161 (24·5%)	66 (30·6%)	
≥18 years	66 (10·0%)	22 (10·2%)	
Student type			
Day	381 (57·9%)	122 (56·5%)	0·95
Boarding	277 (42·1%)	94 (43·5%)	
Religion			
Catholic	234 (35·6%)	65 (30·1%)	0·27
Protestant/Born Again/SDA	249 (37·8%)	81 (37·5%)	
Muslim	173 (26·3%)	68 (31·5%)	
None/Other	2 (0·3%)	2 (0·9%)	
Ethnicity			
Muganda	458 (69·6%)	152 (70·4%)	0·71
Non Muganda	200 (30·4%)	64 (29·6%)	
Primary caregiver			
Mother	340 (51·7%)	111 (51·4%)	0·94
Father	242 (36·8%)	81 (37·5%)	
Self	7 (1·1%)	3 (1·4%)	
Other	69 (10·5%)	21 (9·7%)	
Household size			
0-5 people	204 (31·0%)	64 (29·6%)	0·89
6-7 people	219 (33·3%)	70 (32·4%)	
≥8 people	235 (35·7%)	82 (38·0%)	
Meals eaten previous day			
Three or more	243 (36·9%)	65 (30·1%)	0·17
Two	333 (50·6%)	126 (58·3%)	
One or fewer	82 (12·5%)	25 (11·6%)	
Relative socioeconomic position			
Lowest	221 (33·6%)	72 (33·3%)	0·99
Medium	219 (33·3%)	71 (32·9%)	
Highest	218 (33·1%)	73 (33·8%)	

2.6. Figure: Male participant profile

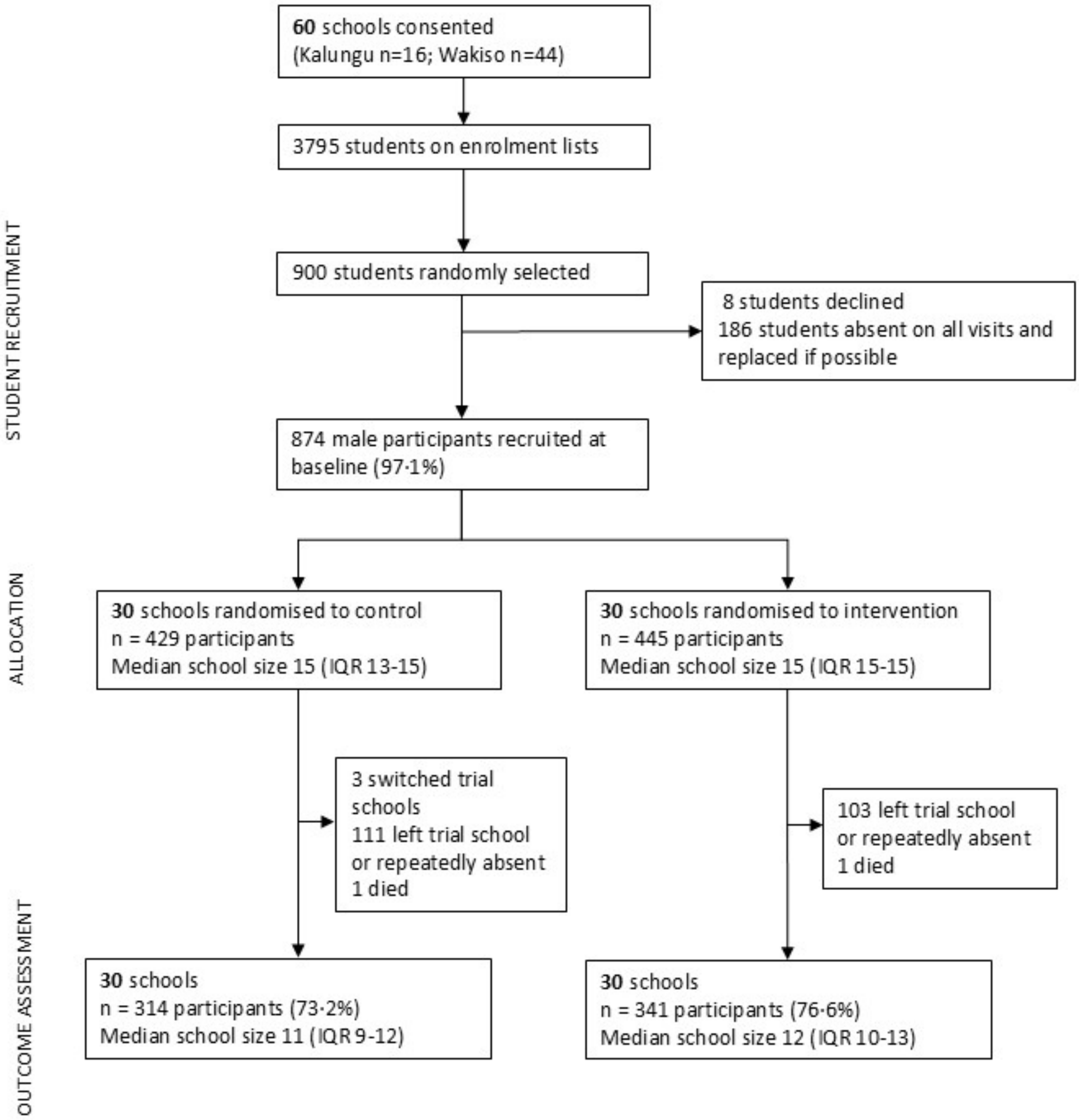

2.7. Figure: Diary sub-study participant profile

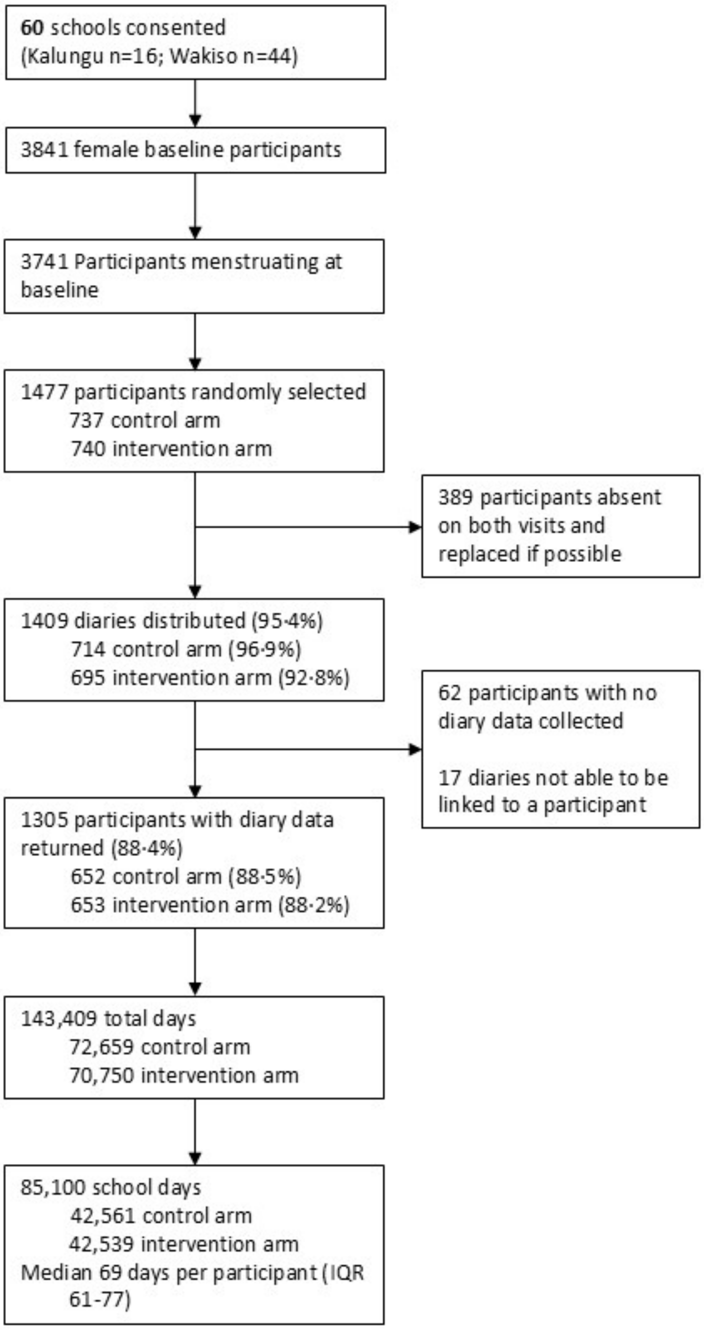

2.8. Figure: Distribution of SAMNS and MPNS scores by arm

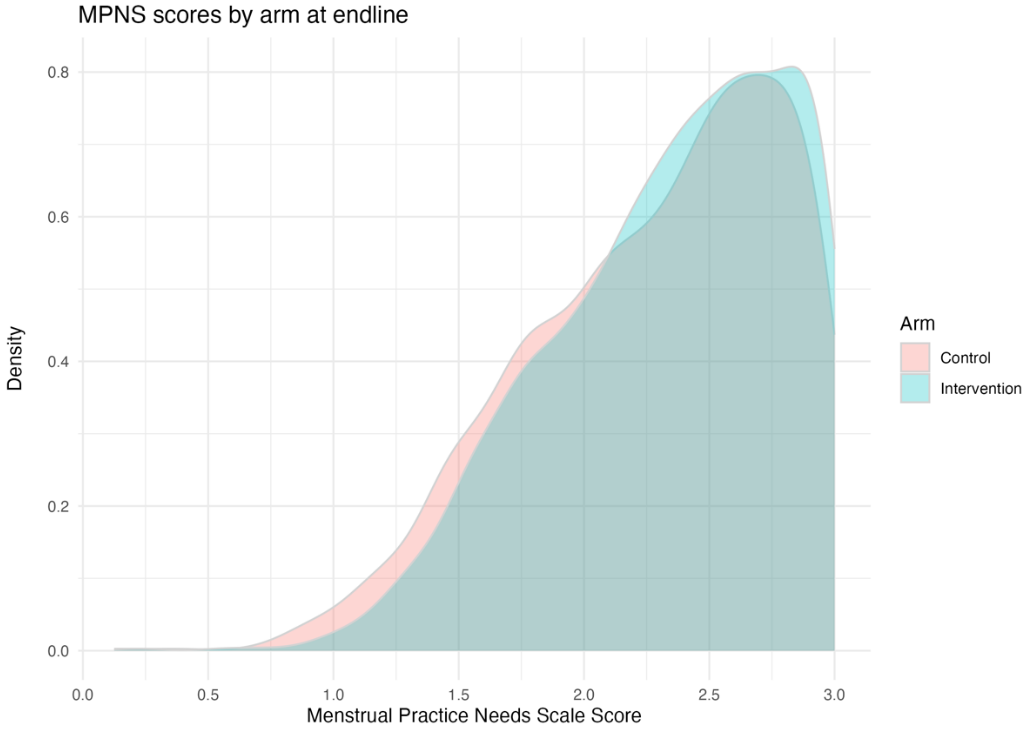

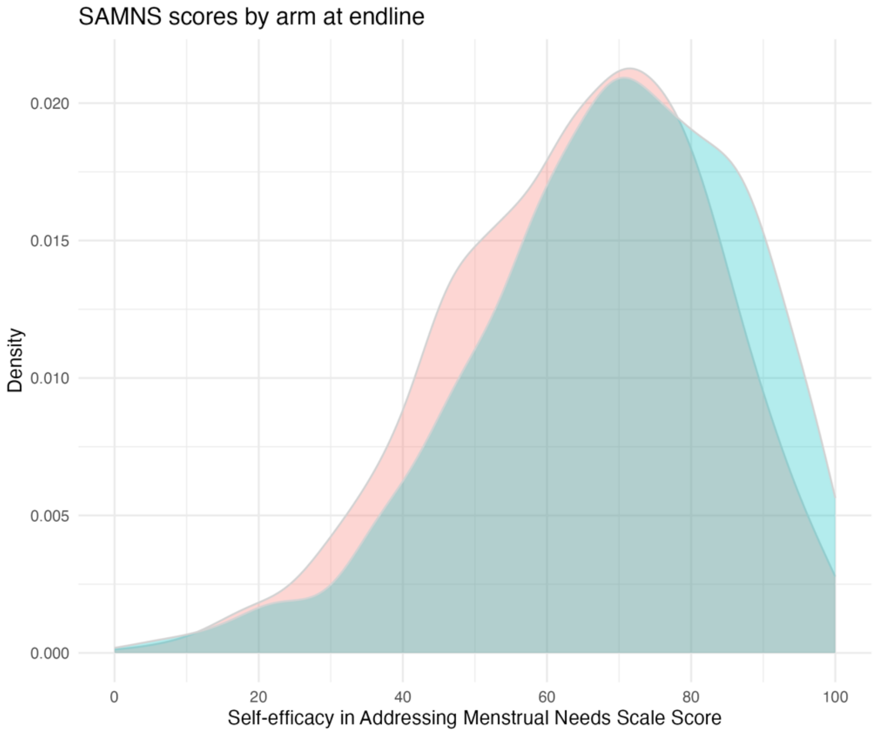

3. Safety reporting

Serious AEs were monitored in both arms and non-serious AEs were monitored in the intervention arm only

3.1. Table: Serious AEs and non-serious AEs

	**Intervention arm (n, %)**	**Control arm (n; %)**	**Risk difference (95% CI)**
**Serious adverse events in female participants**	**N=2093[1]**	**N=2095[1]**	
Psychological distress secondary to rape	1 (not related)	1	
Severe anaemia secondary to excessive vaginal bleeding	1 (possibly related)	0	
**Total SAE in female participants**	**2 (0.10%)**	**1 (0·005%)**	**0·00005 (-0·0011, 0·0021)**
**Serious adverse events in male participants**	**N=445[1]**	**N=429[1]**	
Death due to malaria	1	0	
Death due to injury	0	1	
**Total SAE in male participants**	**1 (0·22%)**	**1 (0·23%)**	**-0·00001 (-0·0064, 0·0063)**
**Non-serious adverse events in female participants (intervention arm only)**	**N=2093[1]**	Not monitored	
Suspected asthma	1 (not related)	··	··
Bacteraemia	1 (not related)	··	··
Urinary tract infection and abnormal vaginal discharge	1 (possibly related)	··	··
**Total non-serious AE**	**3 (0·14%)**	··	

[1] Includes all trial participants consented/assented

4. Additional analyses (pre-specified)

4.1. Table: Intervention effects among female participants in the closed cohort

	Control arm		Intervention arm			
	N	Mean (SE) / n (%)	N	Mean (SE) / n (%)	aMD*, aOR, or aIRR (95% CI)	p value
**Primary outcomes**						
Educational assessment z-score	1375	0·13 (0·02)	1394	0·23 (0·02)	aMD=0·05 (-0·09, 0·19)	0·49
SDQ Total Difficulties Score	1440	10·62 (0·14)	1438	10·61 (0·14)	aMD=-0·06 (-0·56, 0·43)	0·80
**Female secondary outcomes**						
Knowledge score (out of 9)	1440	5·60 (0·04)	1438	6·18 (0·04)	aIRR=1·10 (1·07, 1·14)	<0·001
Attitudes score (out of 3)	1440	1·87 (0·02)	1438	2·26 (0·02)	aIRR=1·22 (1·16, 1·28)	<0·001
Adequate MHM	1343	740 (55·1%)	1313	703 (53·5%)	aOR=0·93 (0·78, 1·11)	0·40
MPNS Score	1344	2·28 (0·01)	1313	2·36 (0·01)	aMD=0·10 (0·05, 0·14)	<0·001
SAMNS score	1347	64·2 (0·49)	1314	68·6 (0·52)	aMD=5·11 (3·38, 6·87)	<0·001
Effective pain management	1133	754 (66·7%)	1074	817 (76·1%)	aOR=1·62 (1·29, 2·03)	<0·001
Symptomatic UTI	1329	297 (22·4%)	1302	231 (17·7%)	aOR=0·74 (0·53, 1·02)	0·07
Confidence in mathematics	1440	1·61 (0·02)	1438	1·61 (0·02)	aMD=-0·005 (-0·06, 0·05)	0·87
Confidence in science	1440	2·00 (0·02)	1438	2·01 (0·02)	aMD=0·02 (-0·03, 0·08)	0·41

aMD = adjusted mean difference, aOR = adjusted odds ratio, aIRR = adjusted incident rate ratio, SMD = standardised mean difference, CI = confidence interval, SDQ = Strengths and Difficulties Questionnaire, MHM = menstrual hygiene management, MPNS = Menstrual Practice Needs Scale, SAMNS = Self Efficacy in Addressing Menstrual Needs Scale, UTI = urinary tract infection.
[1] Adjusted for district, high/low school educational score, and the baseline cluster-level mean of the respective outcome measure where available (not included for symptomatic UTI and school/class absence outcomes; adequate MHM adjusted for use of only adequate material at baseline).

4.2. Table: Sensitivity analyses

		**Primary analysis**[1]		**Cluster-level analysis**[2]		**GEE**[3]	
	Type of effect	Adjusted effect estimate[4] (95% CI)	p-value[5]	Adjusted effect estimate[4] (95% CI)	p-value[5]	Adjusted effect estimate[4] (95% CI)	p-value[5]
**Primary outcomes**							
Educational assessment z-score	aMD	0·05 (-0·11, 0·20)	0·56	0·05 (-0·11, 0·21)	0·56	0·06 (-0·11, 0·23)	0·47
SDQ total difficulties score	aMD	0·05 (-0·40, 0·50)	0·84	0·09 (-0·41, 0·58)	0·73	0·05 (-0·41, 0·52)	0·83
**Female secondary outcomes**							
Knowledge score (out of 9)	aIRR	1·10 (1·07, 1·13)	<0·001	1·12 (1·09, 1·14)	<0·001	1·10 (1·07, 1·13)	<0·001
Attitudes score (out of 3)	aIRR	1·20 (1·14, 1·26)	<0·001	1·24 (1·17, 1·30)	<0·001	1·20 (1·15, 1·25)	<0·001
Adequate MHM	aOR	0·91 (0·76, 1·08)	0·27	0·95 (0·88, 1·04)	0·28	0·92 (0·78, 1·10)	0·35
MPNS Score	aMD	0·09 (0·05, 0·13)	<0·001	0·08 (0·04, 0·13)	<0·001	0·09 (0·05, 0·13)	<0·001
SAMNS score	aMD	4·95 (3·31, 6·59)	<0·001	4·81 (2·84, 6·78)	<0·001	4.95 (3.37, 6.53)	<0·001
Effective pain management	aOR	1.50 (1.25, 1.80)	<0·001	1.16 (1.08, 1.24)	<0·001	1.50 (1.26, 1.79)	<0·001
Symptomatic UTI	aOR	0.74 (0.54, 1.00)	0·08	0.81 (0.61, 1.07)	0·13	0.73 (0.54, 0.98)	0·04
Confidence in mathematics	aMD	0.01 (-0.05, 0.07)	0.77	0.01 (-0.05, 0.07)	0.75	0.004 (-0.06, 0.06)	0.89
Confidence in science	aMD	0.02 (-0.03, 0.08)	0.44	0.01 (-0.05, 0.07)	0.67	0.03 (-0.03, 0·09)	0·34
**Male secondary outcomes**							
Knowledge score (out of 9)	aIRR	1·04 (0·97, 1·11)	0·27	1·03 (0·99, 1·08)	0·16	1·04 (1·00, 1·08)	0·06
Attitudes score (out of 3)	aIRR	1·44 (1·26, 1·64)	<0·001	1·43 (1·22, 1·66)	<0·001	1·44 (1·26, 1·63)	<0·001

aMD = adjusted mean difference, aOR = adjusted odds ratio, aIRR = adjusted incident rate ratio, CI = confidence interval
SDQ = Strengths and Difficulties Questionnaire, MHM = menstrual hygiene management, MPNS = Menstrual Practice Needs Scale, SAMNS = Self Efficacy in Addressing Menstrual Needs Scale, UTI = urinary tract infection.
[1]Primary analysis: Individual-level analysis with random effect for school
[2]Cluster-level analyses: these effects correspond to the cluster-average estimand. In the cluster-level analyses, intervention effects for binary outcomes are estimated as aRRs.

[3]GEE: Generalised estimating equations with an independence correlation structure and robust standard errors to minimise potential bias due to informative cluster size.
[4]Adjusted for district, high/low school educational score, and the baseline cluster-level mean of the respective outcome measure where available (not included for symptomatic UTI and school/class absence outcomes; adequate MHM adjusted for use of only adequate material at baseline).
[5]P-values are obtained by the Wald test for the cluster-level and GEE models

4.3. Table: Intervention effect on the SDQ internalising sub-scale

		**Primary analysis[1]**		**Cluster-level analysis[2]**		**GEE[3]**	
	Type of effect	Adjusted[4] effect estimate (95% CI)	p-value[5]	Adjusted[4] effect estimate (95% CI)	p-value[5]	Adjusted[4] effect estimate (95% CI)	p-value[5]
**Exploratory outcome**							
SDQ internalising sub-scale	aMD	-0·03 (-0·31, 0·25)	0·85	-0·06 (-0·37, 0·25)	0·71	-0·01 (-0·29, 0·26)	0·92

SDQ = Strengths and Difficulties Questionnaire; aMD = adjusted mean difference; CI = confidence interval
[1]Primary analysis: Individual-level analysis with random effect for school
[2]Cluster-level analyses: these effects correspond to the cluster-average estimand. In the cluster-level analyses, intervention effects for binary outcomes are estimated as aRRs.
[3]GEE: Generalised estimating equations with an independence correlation structure and robust standard errors to minimise potential bias due to informative cluster size.
[4]Adjusted for district, high/low school educational score, and the baseline cluster-level mean of the respective outcome measure where available (not included for symptomatic UTI and school/class absence outcomes; adequate MHM adjusted for use of only adequate material at baseline).
[5]P-values are obtained by the Wald test for the cluster-level and GEE models

5. Intra-cluster correlation estimates

The anticipated adjusted ICC used in the sample size calculations was 0·05. The ICC is not meaningful for multi-level count outcomes, so has not been included for knowledge and attitude outcomes.[2]

5.1. Table: Intra-cluster correlation by arm and overall for continuous and binary outcomes

**Outcome**	**Control arm ICC (95% CI)**	**Intervention arm ICC (95% CI)**	**Overall ICC (95% CI)**
**Primary outcomes**			
Educational assessment z-score	0·15 (0·09, 0·24)	0·09 (0·05, 0·15)	0·12 (0·08, 0·18)
SDQ total difficulties score	0·01 (0·001, 0·05)	0·004 (0·00, 0·06)	0·01 (0·00, 0·02)
**Female secondary outcomes**			
Adequate MHM	0·003 (0·00, 0·26)	0·01 (0·00, 0·05)	0·01 (0·00, 0·03)
MPNS Score	0·00 (0·00, 0·00)	0·01 (0·00, 0·05)	0·01 (0·00, 0·03)
SAMNS score	0·01 (0·001, 0·07)	0·003 (0·00, 0·26)	0·01 (0·00, 0·03)
Effective pain management	0·00 (0·00, 0·00)	0·005 (0·00, 0·32)	0·00 (0·00, 0·00)
Symptomatic UTI	0.05 (0.02, 0·13)	0·04 (0·01, 0·12)	0·06 (0·03, 0·11)
Confidence in mathematics	0·01 (0·004, 0·04)	0·01 (0·00, 0·05)	0·01 (0·00, 0·03)
Confidence in science	0·01 (0·001, 0·05)	0·02 (0·01, 0·05)	0·01 (0·01, 0·03)

6. Implementation fidelity

6.1. Table: MENISCUS intervention reported according to the TIDieR Framework

MENISCUS intervention components

1. Menstrual Health (MH) Action Group
2. Puberty education
3. Drama skit
4. Menstrual health kit & training
5. Pain management
6. Water, Sanitation and Hygiene (WASH) improvements

Why: Rationale for each component

1. To facilitate school-ownership of the MENISCUS intervention, and hence that it is implemented well in the school.
2. To strengthen schools' capacity to deliver knowledge of puberty and menstruation
3. To enable a supportive school environment and reduce stigma and teasing
4. To improve menstrual management and provide product choice
5. To improve ability to manage menstrual pain
6. To improve menstrual hygiene management

What: Materials & procedures

1. The WoMena staff training the Action Group will receive a manual and training to deliver the training. The MH Action Group members will receive in-person training in puberty and menstruation and on running an MH Action Group. The MH Action Group members will receive: a T-shirt with the MENISCUS logo, the budget for running the Group, an MH kit and an MH Action Group Charter, guide and action plan to complete. The MH Action Group will receive support by WoMena Uganda after the initial training in follow-up sessions.

2. The schools staff delivering puberty education will receive in-person training on how to deliver the Ministry of Education and Sports' (MoES) training on menstrual health management and a copy of the MoES Training Manual for teachers and other stakeholders on Menstrual Health Management.

3. The drama group members (facilitators and students), will receive two facilitation sessions on menstruation and the drama skit. They will receive an outline of a MH-related drama to be developed into a drama skit and performed at an existing school meeting. A small budget, managed by the MH Action Group, will be available to buy props and supporting materials for the performance and the students taking part. The students taking part will receive a T-shirt with a MENISCUS logo.

4. i) Trained school members ("MENISCUS trainers") including student leaders and prefects to provide peer-support to girls, will receive in-person training on how to deliver menstruation education sessions (a joint session of menstruation for boys and girls and separate girls' and boys' sessions), alongside receiving specially-developed materials to deliver these sessions, flipcharts and a training manual. The trainers will receive a certificate, a T-shirt, and an MH kit.

   ii) S2 students will receive in-person education sessions delivered by the MENISCUS trainers. The students will receive a MENISCUS booklet developed by WoMena to accompany the education sessions. The girls' session includes puberty, how to use the MH kit components, tracking and managing their periods, including managing menstrual pain. Students will receive a MH kit consisting of the AFRIpads "schoolgirl kit" of 5 reusable pads, a towel, soap, two pairs of underwear and MH booklet with a menstrual tracker, plus a menstrual cup and container for girls who consented to the cup. The boys' session will include male puberty, genital hygiene, male circumcision, and attitudes towards girls who are menstruating.

   iii) Female caregivers will receive in person education around menstruation and the use and care of reusable menstrual products (menstrual cup and reusable pads). Caregivers will receive a MH kit of the AFRIpads "standard kit" of 6 reusable pads, a towel, soap, underwear plus a menstrual cup and container to those who consent and are willing to receive the cup.

5. S2 female students will receive i) an information sheet about safe use of paracetamol and ibuprofen, plus information on pain management methods more broadly in the MENISCUS MH booklet; ii)

vouchers to be redeemed for a maximum of 6 tablets per month from the school nurse or designated senior teacher who will be trained in administering these by the MENISCUS clinical officer.

6. Basic improvements to school WASH facilities (installation of locks, repair of broken doors, provision of bins and toilet paper holders fixed to the wall, liquid hand washing soap and water drums).

Who provided

1. The MH Action Group will consist of 6-8 people selected by school management, and will be responsible for implementing and maintaining the intervention. They will be MH champions within the school. MH Action Group members will include a minimum of at least one representative of school management, a senior woman teacher, a student and a parent. The group will receive:
   - 1-day training on menstruation and being a member of the MH Action Group by WoMena Uganda;
   - Follow-up and support from WoMena Uganda facilitators at 3 MH Action Group meetings
2. Up to 5 male and female teachers per school who usually deliver puberty education to secondary students will attend a 2-day training, followed by 1-day training by WoMena Uganda.
3. The drama skit component will involve the drama group in the school, including the drama teacher and students who attend. If there is no active drama club or group in the school the MH Action Group will be encouraged to support the creation of a group. Drama groups, including a drama teacher/facilitator and around 30 students in the school will be invited to 2 facilitation sessions which contain training on menstruation and puberty and introductions to the concept of the drama skit and the script. The group will also have rehearsals observed and supported by WoMena.
4. Approximately 7 school staff and students will be selected as MENISCUS Trainers, responsible for training S2 students in MH and using the MH kit. Selected prefects and student leaders will support the trained staff in training activities and act as peer support in their schools. Training participants will be selected by school management according to specified selection criteria (for example, motivation and agreement to attend trainings and train young people of puberty, menstruation and using reusable menstrual products, as well as having the trust and respect from students). Approximately 8 female caregivers associated with the schools will be selected by school management according to a specified criterion (willing to try products, respected in the community, has a child who attends the school).
5. The school nurse or other designated senior teacher will be trained on safe use and management of paracetamol and ibuprofen by the MENISCUS clinical officer, including how the voucher scheme works.
6. WASH improvements will be made by a contractor employed by MRC/UVRI and LSHTM Uganda Research Unit. The MH Action Group members will be responsible for maintaining the improved WASH facilities.

How: modes of delivery

1. WoMena Uganda will provide face-to-face group training around the MH Action Group and will have in person follow up visits for each school to observe and support.
2. WoMena Uganda will provide face-to-face group training in puberty education to teachers.
3. WoMena Uganda will introduce the drama skit and provide a brief training on menstruation in person, and will attend a rehearsal to give feedback and support in each school.
4. WoMena Uganda will provide face-to-face group training of MENISCUS Trainers, with follow-up in each school to provide support. WoMena Uganda will provide face-to-face group trainings of female caregivers.
5. The MENISCUS Clinical Officer will provide training of school nurses or other designated staff on appropriate analgesic use, and WoMena will provide training on alternative pain management strategies to MENISCUS Trainers.
6. WASH improvements will be made at each school by the contractor.

**Where**<br>All intervention elements will take place at the school or at an event space in the community for the trainings.
**When and how much**<br>All elements will be delivered over the course of a year, following randomisation.<br>1. The training of the MH Action Groups will take place once, in clusters of about 5 intervention schools based on their location. The training will be for one day per cluster of schools. The MH Action Groups following this will run for 8-11 months (depending on when they receive their training). They will have 3 meetings, with one attended by the WoMena Uganda team over this period.<br>2. The training of the puberty educators will be two sessions in each District. The first training will take place approximately 4 weeks ahead of the second training. The first training will last 2 days and the second training will last 1 day. Those trained will then deliver the puberty education to students they teach over the following 8 months.<br>3. Two drama skit facilitation sessions will take place in each school followed by an attendance at one rehearsal. The drama skit facilitation sessions, rehearsals and performance will take place over a period of three months.<br>4. The training of MENISCUS Trainers will take place once with two separate sessions firstly, a 2-day session and a 1-day follow-up session, approximately one month after the initial training session. The MENISCUS Trainers will then deliver this training to students at the school over the following 3 months. The training of female caregivers will take place once in a 1-day session.
**Tailoring**<br>There are plans for adaptations and tailoring based on attendance at the initial trainings and the delivery of the training by the MENISCUS trainers to the S2 students. If there is no attendance at the initial trainings, schools will be offered these trainings at their schools individually unless there are enough schools who did not attend and then another training will be held in a central location. If schools do not deliver the training to S2 students, they will be supported to do so by WoMena Uganda by either delivering the sessions or being present when the school delivers all the sessions.
**How well**<br>This will be evaluated in the Process evaluation (please see details in the protocol paper Table 4)

6.2. Fidelity indicators and definitions

Component	Minimum threshold	Requirements	Corresponding data source
**Action group (AG)**	AG was trained, met, and created an action plan	1. At least 3 people participated in district AG training<br>2. Completed the action plan template<br>3. Held at least 1 AG meeting after WoMena's introductory meeting	1. Training attendance list<br>2. Copy of action plan or self-report and qualitative interview coding<br>3. Copy of minutes from meeting or self-report and qualitative interview coding
**MH training and kit distribution**	All training sessions were delivered to the participants and kits were delivered	1. At least 3 people participated in the district ToT training<br>2. All in-school training sessions took place<br>3. WoMena held at least one kit distribution day	1. Training attendance list<br>2. Implementor records<br>3. Kit distribution records
**Puberty education**	Teachers were trained and delivered at least one session in their school	1. At least 2 relevant (S2/S3 or puberty) teachers participated in both days of the district puberty education training<br>2. Trained teacher delivered at least 1 puberty education module following the district training	1. Training attendance list<br>2. Self-report on school puberty education session tool
**Drama skit**	A school staff member was trained and the skit was performed to the school community	1. At least 1 staff member trained by WoMena to oversee drama skit<br>2. Drama skit performed by students to an audience at the school	1. Implementor records<br>2. Implementor records
**Pain management**	At least one staff member was trained to distribute analgesics, which were typically in stock	1. At least 1 person attended district training on analgesics<br>2. Analgesics were delivered and in stock and accessible at a minimum of 2 of 3 spot checks<br>3. MH training on non-analgesic strategies took place	1. Training attendance list<br>2. Stock check observation database<br>3. Implementor records
**WASH**	All minimum components were delivered, and there is at least one functional and private toilet at endline with a bin, as well as a water source available by the blocks.	1. All necessary materials were delivered/installed by MRC<br>2. At least one single sex female toilet was private and functional at endline<br>3. There was a water source available in or immediately outside the toilet block at endline	1. WASH installation records<br>2. Endline WASH observation<br>3. Endline WASH observation

6.3. Table: Fidelity of intervention implementation by district

Component and indicators	N/non-missing (%) schools meeting criteria		
	Wakiso (n=22)	Kalungu (n=8)	Overall (n=30)
**Action group**	**19/22 (86%)**	**6/7 (86%)**	**25/29 (86%)**
3+ people participated in training	21/22 (95%)	8/8 (100%)	29/30 (97%)
Completed action plan or charter*	19/22 (86%)	6/7 (86%)	25/29 (86%)
1+ additional meeting after introductory meeting	22/22 (100%)	8/8 (100%)	30/30 (100%)
**MH training and kit distribution**	**21/22 (95%)**	**8/8 (100%)**	**29/30 (97%)**
3+ people participated in training	21/22 (95%)	8/8 (100%)	29/30 (97%)
All in-school training took place**	22/22 (100%)	8/8 (100%)	30/30 (100%)
1+ kit distribution day held	22/22 (100%)	8/8 (100%)	30/30 (100%)
**Puberty education**	**18/21 (86%)**	**7/7 (100%)**	**25/28 (89%)**
2+ teachers participated in training	20/22 (91%)	8/8 (100%)	28/30 (93%)
Reported delivery of 1+ puberty education module†	18/21 (86%)	7/7 (100%)	25/28 (89%)
**Drama skit**	**22/22 (100%)**	**8/8 (100%)**	**30/30 (100%)**
1+ staff member trained	22/22 (100%)	8/8 (100%)	30/30 (100%)
Skit performed to a school audience	22/22 (100%)	8/8 (100%)	30/30 (100%)
**Pain management**	**21/22 (95%)**	**8/8 (100%)**	**29/30 (97%)**
1+ staff member participated in training	21/22 (95%)	8/8 (100%)	29/30 (97%)
Analgesics were delivered‡	21/22 (95%)	8/8 (100%)	29/30 (97%)
Training on non-analgesic strategies delivered**	22/22 (100%)	8/8 (100%)	30/30 (100%)
**WASH**	**17/22 (82%)**	**6/8 (75%)**	**23/30 (77%)**
All materials delivered/installed	22/22 (100%)	8/8 (100%)	30/30 (100%)
1+ single sex female toilet private and functional at endline	20/22 (91%)	8/8 (100%)	28/30 (93%)
Water source available in or immediately outside the toilet block at endline	18/22 (82%)	6/8 (75%)	24/30 (80%)
**All fidelity criteria met**	**14/21 (86%)**	**6/7 (86%)**	**20/28 (71%)**

Missing data: n=1 school missing data on completion of action group action plan or charter; n=2 schools missing data on delivery of puberty education

* Schools used the action plan and action group charter templates interchangeably, and self-reported completion or submission of one was considered sufficient for the fidelity indicator

**Following a pre-determined procedure specific to this component, any school that did not attend the central MH training had a follow up visit from implementor WoMena Uganda to ensure participants could safely receive their MH kits.

†While we aimed to capture information on the session content and format, it is possible schools may have conflated this with the MH training due to overlap of content. As a result, this may be an overestimate of the puberty education sessions delivered.

‡Analgesic spot check data were incomplete due to logistical constraints, so this indicator is limited to analgesic stock delivery. Schools which did not attend training could not receive analgesics.

7. Costing methods, detailed results, and sensitivity analysis

7.1. Methods

The cost analysis followed a combination of top-down and bottom-up micro-costing approach based on the Guidelines for Conducting Cost Analyses Of Interventions To Prevent Violence Against Women And Girls (VAWG).[3] The VAWG guidelines are in line with the International Decision Support Initiative (iDSI) guidelines and the Global Health Costing Consortium (GHCC) Reference Case.[4,5] The VAWG guidelines include published and openly-available data collection and entry tools (Microsoft Excel, Seattle, USA), which were adapted for this study .

The MENISCUS intervention was delivered in two phases: start-up and implementation. Activities were reported annually under the two phases. All resource used in intervention schools for the delivery of MENISCUS-related activities were identified, quantified and estimated from the provider's perspective. Start-up costs were annuitized over their expected lifespans of 1.5 year. Discount rates were applied based on official interest rates from the Bank of Uganda. For the start-up period from January 2021 to May 2022, the average interest rate was 7%. For the implementation period, from June 2022 to June 2023, the average interest rate was 11%. .[6]

Resources used were identified for each activity that was conducted in the start-up and implementation phases. Resources that were used across different activities were allocated to each activity using allocation factors . For example, staff time was allocated through interviews detailing the number of activities that each staff conducted during the start-up and implementation phases. Buildings and overheads costs were allocated based on the number of rooms that were occupied and utilized by staff implementing MENSICUS activities This approach ensured that resources were distributed fairly across all aspects of the project. All costs were valued at market prices at the time of resource usage for recurrent costs and at the time of purchase for equipment or capital costs. Economic costs were used to reflect the value of non-financial costs e.g. shadow prices for donated items. All research costs were excluded.

Financial costs were obtained for the period January 2021 to May 2022 for the start-up and June 2022 to June 2023 for the implementation phase, from financial records, project accounts, Monitoring & Evaluation (M&E) registers, and staff time sheets of the implementing partner WoMena, and the MRC office. All data was entered into the VAWG costing tool and stored on an encrypted online folder.

Prices were collected in the local currency (UGX), inflated to year 2023 using GDP deflator from the World Bank and converted to USD using the exchange rate from the Bank of Uganda.[7,8]

Unit costs were calculated by dividing the total financial and economic costs (fixed and variable costs) by the total units of output. The output units were calculated as the number of students enrolled in intervention arm schools (by gender) at baseline and number of schools that received the intervention.

7.2. Table: Activities, resource use and costing sources for each cost component of costing analysis

	Activities per phase	Resources used	Costing sources
**Start Up Phase**	Planning workshop<br>Stakeholder engagement workshop<br>Procurement of equipment<br>Training<br>Exploratory visits<br>Development of training manual<br>Procurement of visible materials	Staff salaries<br>Transport refund<br>Meals (lunch, tea, water)<br>Vehicle fire<br>Printing costs<br>Stationery<br>Laptops<br>Furniture<br>T-shirts<br>Fuel<br>Trainer fees	Project Activity reports<br>Project financial accounts<br>Asset registry<br>Monitoring & Evaluation (M&E) registers<br>Staff time sheets
**Implementation Phase**	Introductory meetings<br>Procurement of Menstrual Health Kits<br>Training<br>Action group meetings<br>Puberty Education<br>Distribution of MHM kits<br>Pain management<br>Implementation of a drama skit<br>Water, Sanitation and Hygiene (WASH)	Staff salaries<br>Fuel<br>Equipment and furniture<br>Stationery<br>Meals (lunch, tea, water)<br>Trainer fees<br>Menstrual Health Kits<br>Per-diem<br>Accommodation<br>Transport<br>Venue hire<br>Procurement of pain killers<br>Printing costs<br>WASH improvements materials	Project Activity reports<br>Project financial accounts<br>Meeting minutes<br>Monitoring & Evaluation (M&E) registers<br>Staff time sheets

7.3. Table: Cost of setting up and running the MENISCUS intervention

	Resources used	US$	% of total cost
**Start Up Phase**	Equipment	1,114	0.50
	Buildings: Spaces	2,861	1.29
	Buildings: Furniture	66	0.03
	Salaried Staff	31,384	14.11
	Supplies*	2,573	1.16
	Building Utilities & Maintenance	1,055	0.47
	Transport: Public Transportation/Rental	1,316	0.59
	Per Diems and Allowances	621	0.28
	**Subtotal:**	**40,990**	**18.42**
**Implementation Phase**	Equipment	4,641	2.09
	Buildings: Spaces	5,740	2.58
	Buildings: Furniture	783	0.35
	Salaried Staff	64,922	29.18
	Supplies*	73,337	32.96
	Building Utilities & Maintenance	1,645	0.74
	Transport: Vehicle Operations (fuel)	2,763	1.24
	Transport: Public Transportation/Rental	16,859	7.58
	Per Diems and Allowances	10,813	4.86
	**Subtotal:**	**181,503**	**81.58**
	**Total:**	**222,493**	**100.00**

*Supplies include: menstrual reusable pads, menstrual cups and other menstrual-related products, educational material, pain killers, WASH fittings, stationery, accommodation, meals and refreshments, financial transaction costs.

7.4. Table: Implementation cost per intervention component (USD)

Component	Implementation cost (USD)	% of total
Cross-cutting activities	4,759	2.6%
Puberty education	11,383	6.3%
Drama skit	19,939	11.0%
Menstrual health kit	65,745	36.2%
Pain relief	6,416	3.5%
Water, sanitation, and hygiene facilities	21,047	11.6%
Action groups	29,484	16.2%
Management and administration	*22,729*	*12.52%*

7.5. Table: Total cost per beneficiary

Unit	Output units	Unit cost (UGX)	Unit cost (USD)
Total annualized cost per Senior 2 student (implementation cost only*)	4,120	161,332	44
Total annualized cost per female Senior 2 student (implementation cost only)	2,138	310,892	85
Total incremental cost per school (implementation cost only)	30	22,156,235	6,050
Total annualized cost per Senior 2 student (start up and implementation cost**)	4,120	197,766	54
Total annualized cost per female Senior 2 student (start up and implementation cost)	2,138	381,102	104
Total incremental cost per school (start up and implementation cost)	30	27,159,884	7,416

* Total implementation cost = 664,687,046 UGX / 181,503 USD
** Total start up and implementation cost = 814,796,517 UGX / 222,493 USD (Unit costs have been rounded)

7.6. Sensitivity analysis

Univariate sensitivity analysis was conducted on selected key parameters: 1) lifespan of start-up activities; 2) shadow price of donated item (menstrual cup); 3) discount rate; 4) exchange rate.

Doubling the lifespan of the start-up activities from 1.5 years to 3 years reduced total economics costs by 48 %.

In the study, we used a shadow price of US$ 7.25 for the donated menstrual cups. Changing the shadow price of the menstrual cup to US$ 20 (the most expensive cup available in Uganda), increased the implementation cost per S2 female student reached by 4%.

Changing the discount rate from 11% to 3% and to 7% had a negligible impact on the total costs. There was a 1% change in the total costs when the discount rate was changed from 11% to 3% and less than a percentage change when the discount rate was changed to 7%.

Changing the exchange rate from the lowest and highest in the year 2023, increased the total economic cost by 2% and decreased the total economic costs by 4% respectively.

7.7. Cost comparison

To compare the MENISCUS intervention with a similar study in rural Kenya that assessed the cost of providing menstrual cups and sanitary pads to schoolgirls, we summed the reported annual unit costs from the Kenya study: menstrual health cup (US$3.27), sanitary pads (US$24), soap for hygiene (US$1.50), training of nurses (US$0.17), and puberty education (US$1.77). The costs were adjusted for inflation to 2023 US Dollars, resulting in an annual unit cost of US$34.

References

1 Benjamini Y, Hochberg Y. Controlling the False Discovery Rate: A Practical and Powerful Approach to Multiple Testing. *Journal of the Royal Statistical Society: Series B (Methodological)* 1995; **57**: 289–300.

2 Austin PC, Stryhn H, Leckie G, Merlo J. Measures of clustering and heterogeneity in multilevel Poisson regression analyses of rates/count data. *Statistics in Medicine* 2017; **37**: 572.

3 Ferrari, Giulia, Torres-Rueda, Sergio, Michaels-Igbokwe, Christine, Watts, Charlotte, Vassall, Anna. Guidelines for conducting cost analyses of interventions to prevent violence against women and girls in low- and middle-income settings. What Works. 2018; published online Dec 21. https://www.whatworks.co.za/resources/project-resources/item/557-guidelines-for-conducting-cost-analyses-of-interventions-to-prevent-violence-against-women-and-girls-in-low-and-middle-income-settings (accessed May 31, 2024).

4 Wilkinson T, Sculpher MJ, Claxton K, *et al.* The International Decision Support Initiative Reference Case for Economic Evaluation: An Aid to Thought. *Value in Health* 2016; **19**: 921–8.

5 DeCormier Plosky W, Bollinger LA, Alexander L, *et al.* Developing the Global Health Cost Consortium Unit Cost Study Repository for HIV and TB: methodology and lessons learned. *African Journal of AIDS Research* 2019; **18**: 263–76.

6 Bank of Uganda interest rates. https://www.bou.or.ug/bouwebsite/Statistics/.

7 World Bank Group. World Development Indicators: GDP deflator (base year varies by country). 2024. https://data.worldbank.org/indicator/NY.GDP.DEFL.ZS.

8 Bank of Uganda exchange rates. https://www.bou.or.ug/bouwebsite/ExchangeRates/index.html.
