# Supplementary material for: Effects and costs of a multi-component menstrual health intervention (MENISCUS) on mental health problems, educational performance, and menstrual health in Ugandan secondary schools: an open-label, school-based, cluster-randomised controlled trial
Source: Lancet Glob Health. 2025 Apr 24;13(5):e888–99. doi: 10.1016/S2214-109X(25)00007-5 (PMC12041187; doi:10.1016/S2214-109X(25)00007-5)
Supplement: Equitable Partnership Declaration [file mmc2.pdf]

THE LANCET
Global Health

Supplementary appendix 2

This Equitable Partnership Declaration (EPD) was submitted by the authors, and we reproduce it as supplied. It has not been peer reviewed. *The Lancet*'s editorial processes have not been applied to the EPD.

Supplement to: Nelson KA, Lagony S, Kansiime C, et al. Effects and costs of a multi-component menstrual health intervention (MENISCUS) on mental health problems, educational performance, and menstrual health in Ugandan secondary schools: an open-label, school-based, cluster-randomised controlled trial. *Lancet Glob Health* 2025; **13:** e888–99.

Equitable Partnership Declaration

Researcher considerations

1. Please detail the involvement that researchers who are based in the region(s) of study had during a) study design; b) clinical study processes, such as processing blood samples, prescribing medication, or patient recruitment; c) data interpretation; and d) manuscript preparation, commenting on all aspects. If they were not involved in any of these aspects, please explain why.

   This question is intended for international partnerships; if all your authors are based in the area of study, this question is not applicable.

   This should include a thorough description of their leadership role(s) in the study. Are local researchers named in the author list or the acknowledgements, or are they not mentioned at all (and, if not, why)? Please also describe the involvement of early career researchers based in the location of the study. Some of this information might be repeated from the Contributors section in the manuscript. Note: we adhere to ICMJE authorship criteria when deciding who should be named on a paper.

**a) Study design:**<br>Twenty of the named co-authors are based in Uganda. Of these, three (CK, CA, FM) had substantial input into the funding proposal along with UK-based researchers (HAW (PI), BT, CT, GG, JJ, CB, JAS). The other Ugandan co-authors were recruited after the trial was funded, and provided input into the protocol and subsequent amendments to the study design.
**b) Clinical study processes:**<br>Recruitment and data collection procedures were led by Ugandan-based Research Team Leads (RBak, CBa, RN, PN, BN, DN, EO, TS, and AT) under the leadership of CK and SL (Ugandan-based Trial Managers). WoMena Uganda staff (SNa, AA, SB), a Ugandan-based NGO, led the intervention delivery.
**c) Data interpretation:**<br>The data were curated by RBat, LM, and CBa (based in Uganda), with KT, KAN, and HAW (based in the UK). LM, KAN, and HAW undertook the formal analyses. Findings were discussed and interpreted by the trial team during a week-long workshop in Entebbe, Uganda.
**d) Manuscript preparation:**<br>KAN (based in the UK) led the writing of the paper, in collaboration with SL (based in Uganda) and HAW (UK). KAN and SL are early career researchers. All authors reviewed and commented on manuscript drafts.

2. Were the data used in your study collected by authors named on the paper, or have they been extracted from a source such as a national survey? ie, is this a secondary analysis of data that were not collected by the authors of this paper. If the authors of this paper were not involved in data collection, how were data interpreted with sufficient contextual knowledge?

   The Lancet Global Health *believe contextual understanding is crucial for informed data analysis and interpretation.*

The data were collected by the authors named on this paper.

3. How was funding used to remunerate and enhance the skills of researchers and institutions based in the area(s) of study? And how was funding used to improve research infrastructure in the area of study?

   Potentially effective investments into long-term skills and opportunities within institutions could include training or mentorship in analytical techniques and manuscript writing, opportunities to lead all or specific aspects of the study, financial remuneration rather than requiring volunteers, and other professional development and educational opportunities.

   Improvements to research infrastructure could be funding of extended trial designs (such as platform trials) and use of master protocols to enable these designs, establishment of long-term contracts for research staff, building research facilities, and local control of funding allocation.

Skills:
The funding provided to the MENISCUS trial was used to part or fully employ researchers based in the study region through the MRC/UVRI and LSHTM Uganda Research Unit or the NGO WoMena Uganda. Throughout the study, Ugandan team members participated in informal and formal skills training on research ethics, qualitative and quantitative methods and analysis, manuscript writing skills, use of referencing software through structured in-person workshops for the MENISCUS team based in Uganda, led by Ugandan and UK-based researchers.

During the course of the trial, two of the Ugandan researchers successfully completed MSc with LSHTM (CB; PN) with mentorship from the Co-Is.

Funding from the trial was used to support Ugandan-based researchers to attend and present findings at international conferences in Sweden, the UK and South Africa.

Research infrastructure:
The trial provided an opportunity for innovation in research including the first use of video assent at the MRC/UVRI Uganda research unit, and use of optical mark recognition (OMR) to scan paper-completed diairies. The Ugandan-based team now was centrally involved in both innovations and will use their expertise in future studies.

4. How did you safeguard the researchers who implemented the study?

   Please describe how you guaranteed safe working conditions for study staff, including provision of appropriate personal protective equipment, protection from violence, and prevention of overworking.

The trial started during the 2020/2021 COVID-19 pandemic and we ensured the use of appropriate personal protective equipment such as gloves, masks and social distancing, tailored to the risks associated with study activities. We implemented safety protocols in line with best

practices, the policy of the MRC/UVRI and LSHTM Uganda Research Unit, and regulatory standards. This included standard operating procedures for handling potentially hazardous materials (urine samples for UTI testing). When staff had weekend fieldwork activities, they were encouraged to take time off in lieu. Each team had a designated safeguarding lead to ensure that trial participants who raised concerns were guided to appropriate agencies for counselling, for their benefit and to avoid the team being put in a position of managing cases beyond their training and expertise.

Benefits to the communities and regions of study

5. How does the study address the research and policy priorities of its location?

   How were the local priorities determined and then used to inform the research question? Who decided which priorities to take forward? Which elements of the study address those priorities?

The initial idea for a trial to improve menstrual health in secondary schools in Uganda came from a Ugandan doctor, Dr George Miiro, who led the initial formative work in 2015. At that time, the Ugandan Government had shown the political will to improve menstrual health, for example by forming a National Menstrual Hygiene Management (MHM) Steering Committee, holding the first international MHM conference in 2014 and celebrating International MHM Day each year.

Prof Helen Weiss took over as PI on the studies upon Dr Miiro's death in November 2016. From our first formative research to the end of the trial, we conducted stakeholder workshops which included representatives from the Ministry of Education and Sports, Ministry of Heath and the District Education Officers. The intervention was designed to be sustainable and integrated within the Ugandan school system.

6. How will research products be shared in the community of study?

   For instance, will you be providing written or oral layperson summaries for non-academic information sharing? Will study data be made available to institutions in the region(s) of study? The Lancet Global Health *encourages authors to translate the summary (abstract) into relevant languages after paper editing; do you intend to translate your summary?*

We disseminated results of the trials to the Ministry of Education and Sports, and to the Uganda National Exam Board in May 2024, and conducted a District-level dissemination for representatives from schools in the trial in June 2024. At these meetings, we shared results of the trials and solicited opinion on which elements of the study could have been done differently and how the intervention could be scaled up. We also created a video which is freely and publicly available, to share results with the wider community (MENISCUS Trial | LSHTM).

The study data can be accessed on request, including by Ugandan institutions. The de-identified individual participant data that underlie the results reported in this article are available indefinitely on request from the London School of Hygiene & Tropical Medicine Data Compass at https://doi.org/10.17037/DATA.00003822, along with the codebook, informed consent

documents, and qualitative interview guides. Participants gave informed consent for their data to be published after de-identification.

7. How were individuals, communities, and environments protected from harm?

a) *How did you ensure that sensitive patient data was handled safely and respectfully? Was there any potential for stigma or discrimination against participants arising from any of the procedures or outcomes of the study?*

Personal data were handled in compliance with GDPR. Individual observations are identified by unique ID numbers. Documents linking ID numbers to participant names are kept separately and securely.

b) *Might any of the tests be experienced as invasive or culturally insensitive?*

Based on formative and pilot work and consultations with community advisory boards, no study procedures were considered invasive or culturally insensitive to our knowledge.

c) *How did you determine that work was sensitive to traditions, restrictions, and considerations of all cultural and religious groups in the study population?*

Ahead of the study, the proposed activities were discussed with the headteachers, and groups of potential trial participants as part of a rapid assessment that has been published (DOI: https://doi.org/10.1371/journal.pgph.0002665). Prior to recruitment we held meetings to discuss the proposed work with parents/guardians and the study participants to address their questions. From our earlier work, we knew that use of the menstrual cup was potentially sensitive due to concerns that it caused girls to lose their virginity. We therefore gave all parents/students the option to participate in the trial but not consent to receive a menstrual cup.

d) *Were biowaste and radioactive waste disposed of in accordance with local laws?*

Waste from urine samples was disposed of in accordance with the MRC/UVRI & LSHTM Uganda Research Unit's regulations and procedures.

e) *Were any structures built that would have impacted members of the community or the environment (such as handwashing facilities in a public space)? If so, how did you ensure that you had appropriate community buy-in?*

As part of the intervention, we improved school water and sanitation facilities, including fixing broken toilet doors, installing locks, and providing handwash facilities near the toilet blocks. This was discussed with the schools during the rapid assessment prior to the intervention.

f) *How might the study have impacted existing health-care resources (such as staff workloads, use of equipment that is typically employed elsewhere, or reallocation of public funds)?*

As part of the intervention, school nurses in intervention schools were trained to distribute analgesics on request from participants. This was already part of their responsibilities and to our knowledge this did not have a substantial impact on their workloads.

8. Finally, please provide the title (eg, Dr/Prof, Mr/Mrs/Ms/Mx), name, and email address of an author who can be contacted about this statement. This can be the corresponding author.

Name: Prof Helen A Weiss
Email: helen.weiss@lshtm.ac.uk
